# Computational and NMR studies of RNA duplexes with an internal pseudouridine-adenosine base pair

**DOI:** 10.1038/s41598-019-52637-0

**Published:** 2019-11-07

**Authors:** Indrajit Deb, Łukasz Popenda, Joanna Sarzyńska, Magdalena Małgowska, Ansuman Lahiri, Zofia Gdaniec, Ryszard Kierzek

**Affiliations:** 1 Institute of Bioorganic Chemistry, Polish Academy of Sciences, Noskowskiego 12/14, 61-704 Poznan, Poland; 2 Department of Biophysics, Molecular Biology & Bioinformatics, University of Calcutta, Kolkata, 700009 West Bengal India; 3 NanoBioMedical Centre, Adam Mickiewicz University, Umultowska 85, 61-614 Poznan, Poland; 4 Present Address: Department of Biophysics, University of Michigan, 930 North University Avenue, Ann Arbor, Michigan 48109 USA

**Keywords:** RNA, Computational biophysics, Molecular dynamics

## Abstract

Pseudouridine (Ψ) is the most common chemical modification present in RNA. In general, Ψ increases the thermodynamic stability of RNA. However, the degree of stabilization depends on the sequence and structural context. To explain experimentally observed sequence dependence of the effect of Ψ on the thermodynamic stability of RNA duplexes, we investigated the structure, dynamics and hydration of RNA duplexes with an internal Ψ-A base pair in different nearest-neighbor sequence contexts. The structures of two RNA duplexes containing 5′-GΨC/3′-CAG and 5′-CΨG/3′-GAC motifs were determined using NMR spectroscopy. To gain insight into the effect of Ψ on duplex dynamics and hydration, we performed molecular dynamics (MD) simulations of RNA duplexes with 5′-GΨC/3′-CAG, 5′-CΨG/3′-GAC, 5′-AΨU/3′-UAA and 5′-UΨA/3′-AAU motifs and their unmodified counterparts. Our results showed a subtle impact from Ψ modification on the structure and dynamics of the RNA duplexes studied. The MD simulations confirmed the change in hydration pattern when U is replaced with Ψ. Quantum chemical calculations showed that the replacement of U with Ψ affected the intrinsic stacking energies at the base pair steps depending on the sequence context. The calculated intrinsic stacking energies help to explain the experimentally observed sequence dependent changes in the duplex stability from Ψ modification.

## Introduction

RNA features a vast collection of chemically and structurally diverse posttranscriptionally modified nucleosides. Pseudouridine (Ψ) was the first such modification discovered^1^ and is the most abundant. Ψ has historically been viewed as an attribute of stable noncoding RNAs including tRNA, rRNA and snRNA^2^. The development of next-generation sequencing methods for detection and mapping of RNA modifications revealed that Ψs are also abundant in mRNA and long noncoding RNA. The number of Ψs in mRNA was found to be dynamically regulated in response to cellular conditions^3–6^. Recent studies have discovered that Ψ participates in the control of various layers of gene expression regulation^7,8^. Research on Ψ, whose role ranges from fine-tuning to being functionally essential to the target RNA, is gaining renewed interest^9–11^.

**Figure 1:**
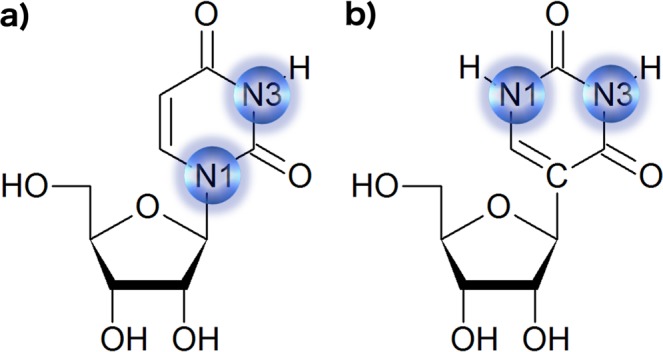
Chemical structure of (**a**) uridine and (**b**) pseudouridine.

Pseudouridine is an isomer of the nucleoside uridine in which the uracil is attached to the sugar via the C5-C1′ instead of the N1-C1′ glycosidic bond (Fig. 1). Compared with uridine, Ψ has an additional hydrogen bond donor (N1 imino proton) and the same Watson-Crick base pairing property. In understanding the mechanism of Ψ functionality, significant contributions have been made by structural and thermodynamic studies. Previous studies reported that the presence of Ψ mainly modulates local RNA conformation and flexibility and generally is not associated with major structural changes^2^. In most of the structures studied so far, Ψ contributed to the structural stability^2^. The impact of the Ψ modification depends on the RNA secondary structure context (stems or loops). Early NMR studies revealed that the presence of Ψ in single strand RNA favors the formation of the C3′-*endo* conformation and promotes base stacking, thus stabilizing RNA structures^12^. In RNA hairpins Ψ stabilizes base pairing and stacking interactions. In particular, in the anticodon-stem loop of tRNA, Ψ39 is involved in the stabilization of the A31-Ψ39 base pair at the interface between the anticodon loop and stem region^13–16^. The consecutive Ψ38 and Ψ39 residues in tRNA^His^ provide stacking stabilization for bases on the 3′ side of the tRNA anticodon loop^17^. Also, the conserved Ψ modifications in helix 69 (H69) from large subunit ribosomal RNA have been shown to modulate the structure and conformational behavior of the stem-loop region by stabilizing the loop-closing base pair and promoting base stacking in the 3′ half of the loop^18,19^ and an additional A-Ψ base pair within the loop^20^. The UV melting data revealed that the Ψ residues in the stem region provided thermodynamic stabilization to the RNA, whereas individual Ψs located in the loop region contributed to a slight destabilization^21,22^. Moreover, structural studies of the P6.1 hairpin derived from the human telomerase RNA activation domain revealed that two Ψ modifications located in the loop increased both base-stacking and hydrogen-bonding interactions within the loop compared with the unmodified construct, resulting in higher thermodynamic stability. Here, the insertion of two Ψs in the loop increased the RNA thermodynamic stability more than the insertion of two Ψs in the stem^23^. The presence of Ψ modification within the branch-site recognition region of splicesomal U2 snRNA induces a bulged conformation of the branch-point adenosine^24,25^. Replacing uridines with Ψ in RNAs containing toxic CUG or CCUG repeats that form helical conformations induced structural stabilization^26^. Furthermore, Ψ is known to modulate codon-anticodon interactions between mRNA and tRNA^17^ allowing purine-purine base pairing^27,28^. The effect of Ψ modification on structure and thermodynamic stability is commonly attributed to the stabilization of the C3′-*endo* conformation, increased base stacking properties^12^, and the presence of an additional N1 H-bond donor that coordinates structural water molecules^29,30^, and all these components are considered to be interdependent^17^.

We previously reported the influence of pseudouridylation on the thermodynamic stability of RNA duplexes with Ψ modifications where the Ψ was located at various positions and formed base pairs with A, G, U and C^31^. For RNA duplexes with central Ψ-A base pairs, we determined the influences of A-U and G-C flanking base pairs on the thermodynamic stabilities. Our results indicated that the enhancement in the duplex stability from replacing U-A with Ψ-A depends on the sequence context. When U was replaced with Ψ in the duplex with the 5′-CΨG/3′-GAC motif, we observed greater enhancement in the duplex stability (2.43 kcal/mol) than those in duplexes with other motifs (up to 0.7 kcal/mol). Moreover, in the duplex with the 5′-AΨU/3′-UAA motif, the replacement of U with Ψ stabilized the duplex 3 kcal/mol less than what was predicted using the available nearest-neighbor parameters^32^.

In this work, we determined the NMR structures of two 9-mer RNA duplexes with 5′-GΨC/3′-CAG and 5′-CΨG/3′-GAC motifs, to examine whether structural factors could be responsible for the differences observed in the increment of thermodynamic stabilities. To further elucidate how the replacement of the middle U-A base pair with Ψ-A in different sequence contexts influences the structure, dynamics and hydration of RNA duplexes, we performed a systematic study using molecular dynamics (MD) simulations. We carried out MD simulations in explicit solvent for all duplexes with internal Ψ-A base pairs studied in previous work^31^, namely, duplexes with 5′-GΨC/3′-CAG, 5′-CΨG/3′-GAC, 5′-AΨU/3′-UAA and 5′-UΨA/3′-AAU motifs and their unmodified counterparts. To understand how the replacement of U with Ψ changes the stacking interactions, we applied high-level *ab initio* methods and performed calculations for eight unique base pair steps for the RNA structures derived from MD simulations.

## Results

The RNA duplexes considered in this study are listed in Table 1.

**Table 1:** List of the Ψ-modified RNA duplexes and their unmodified counterparts studied in this work.

Duplex name	Sequence	Method		Duplex name	Sequence	Method
		NMR	MD			MD
duplex-GΨC	5′-UCAGΨCAGU-3′ 3′-AGUCAGUCA-5′	✓	✓	duplex-GUC	5′-UCAGUCAGU-3′ 3′-AGUCAGUCA-5′	✓
duplex-CΨG	5′-UCACΨGAGU-3′ 3′-AGUGACUCA-5′	✓	✓	duplex-CUG	5′-UCACUGAGU-3′ 3′-AGUGACUCA-5′	✓
duplex-AΨU	5′-UCAAΨUAGU-3′ 3′-AGUUAAUCA-5′		✓	duplex-AUU	5′-UCAAUUAGU-3′ 3′-AGUUAAUCA-5′	✓
duplex-UΨA	5′-UCAUΨAAGU-3′ 3′-AGUAAUUCA-5′		✓	duplex-UUA	5′-UCAUUAAGU-3′ 3′-AGUAAUUCA-5′	✓

Underlined are three nucleotides in the middle of the first strand of the duplexes. Sequences of the modified or unmodified duplexes differ only in the base pairs flanking the central Ψ-A and U-A, respectively.

### NMR study of duplex-GΨC and duplex-CΨG

**Figure 2:**
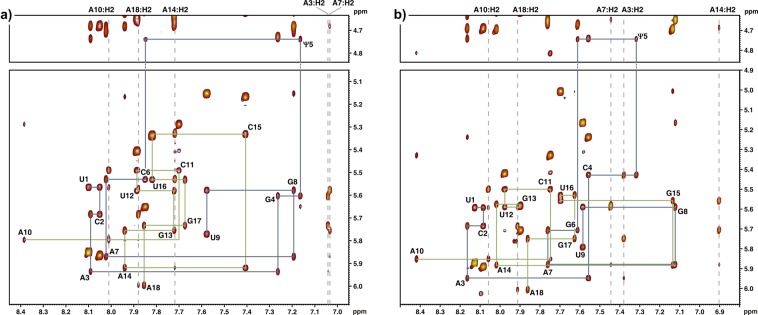
Fingerprint (H6/H8-H1′) regions of the 400 ms NOESY spectra recorded in D_2_O at 25 °C. The sequential connectivities are traced with either a blue or a green line for both strands of the (**a**) duplex-GΨC and (**b**) duplex-CΨG.

Analysis of the 2D NOESY spectra (recorded in D_2_O) used for nonexchangeable proton assignment revealed NOE connectivity typical of A-form geometry. Expanded contour plots of the NOESY spectra corresponding to the interactions between the base H6/H8 and H1′/H5 protons with sequential pathways traced for both duplexes are shown in Fig. 2. For the Ψ residues at position 5 (Ψ5), characteristic upfield shifts of the H1′ resonances relative to the remaining H1′ resonances were observed (Supplementary Tables S1, S3). This tendency is a signature of Ψ modification and is consistent with previous NMR reports regarding pseudouridine-containing RNAs^15,23,33^. Similar to ^1^H, the ^13^C chemical shifts of C1′-Ψ5 differ significantly from the ranges observed for the other anomeric carbon resonances (Supplementary Tables S2, S4). The upfield shift of the H1′ and C1′ resonances in the Ψ residues can be explained by the less electronegative C5 replacing the N1 atom in the N-glycosidic bond position^12,30^. The resonance assignments of the duplex-GΨC and -CΨG are listed in Supplementary Tables S1–S4.

**Figure 3:**
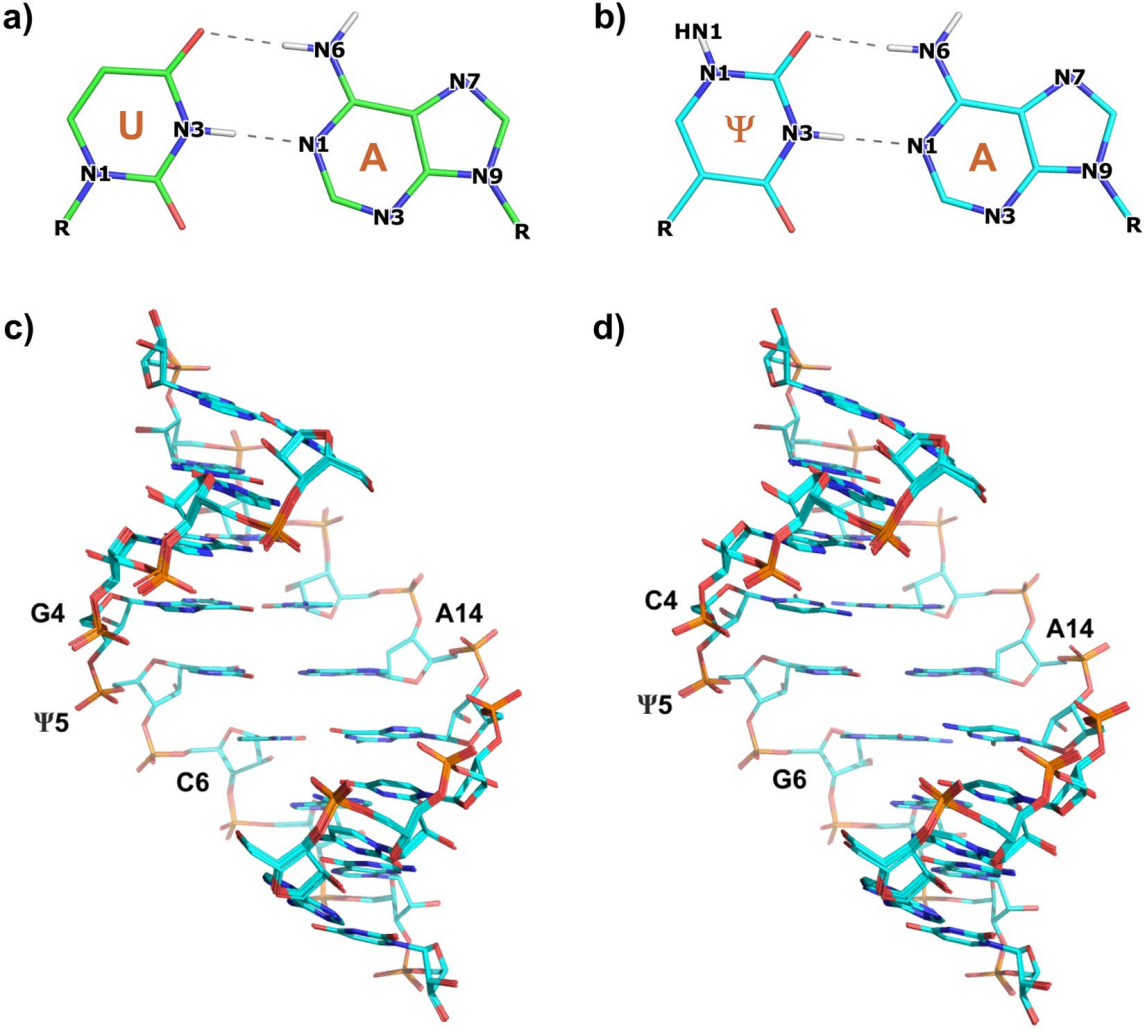
Base pairing of (**a**) uridine and (**b**) pseudouridine with adenosine. Superposition of the ten lowest-energy solution NMR structures: (**c**) duplex-GΨC, (**d**) duplex-CΨG.

The NMR structures of the GΨC- and CΨG-duplexes were solved on the basis of NMR observables (Table 2) using the restrained molecular dynamics approach described in the Methods section. The final set of 10 structures showed that both duplexes form well-defined structures with overall RMSD for heavy atoms within 0.20 Å and 0.21 Å for duplex-GΨC and duplex-CΨG, respectively (Table 2, Fig. 3). To explore the conformational differences between both duplexes, we calculated the geometric parameters^34^. The results of the Curves+ analysis indicated that the duplexes adopted typical A-form conformations, with helical parameters and dihedral angles (backbone, sugar and glycosidic) that fall within the distributions of canonical A-RNA duplexes^35^. This result implied the *anti*-orientation of all the nucleobases and C3′-*endo* sugar ring conformation with calculated average pseudorotation angles and puckering amplitudes of approximately 11° and 42°, respectively. The calculated C1′-C1′ distances averaged over all the base pairs in all the models were 10.75 ± 0.05 Å for both duplexes. A detailed comparison of the two duplexes with the use of rigid body parameters that describe the geometry of the base pairs and sequential base pair steps (Supplementary Tables S5, S6) showed that the change of the 5′ versus 3′ order of the G-C and C-G Watson-Crick base-pairs adjacent to the central Ψ-A pair did not affect the local structure.

**Table 2:** Restraints and structural refinement statistics for ten structures of RNA duplexes with Ψ.

Restraints statistics		
	Duplex-GΨC 6I1W	Duplex-CΨG 6I1V
**No. of restraints**		
Total	657	631
Distance restraints, excluding hydrogen bonds	335	310
Intra-residue	206	189
Sequential residues	119	114
Long range	10	7
Hydrogen bond	54	54
Dihedral restraints	178	177
Chirality restraints	90	90
**Structural refinement statistics**		
Violations		
Average No. of violations (<0.1 Å)	21.70 (0.48)	10.10 (0.32)
Average No. of violations (0.1–0.2 Å)	2.00 (0.00)	<0.01 (0.00)
Maximum violation (Å)	0.12 (0.00)	0.07 (0.00)
Mean torsion penalty (kcal/mol)	<0.01 (0.00)	<0.01 (0.00)
Mean distance penalty (kcal/mol)	1.39 (0.01)	0.17 (0.00)
Mean deviations from idealized geometry		
Bonds (Å)	0.01 (0.00)	0.01 (0.00)
Angles (deg)	2.46 (0.00)	2.45 (0.01)
Maximum RMSD (Å) for heavy atoms	0.20	0.21

Standard deviations over 10 structures are in parentheses.

### Molecular dynamics: Replacement of U with Ψ has only subtle effects on the duplex structures

To evaluate the effects of the Ψ modification on the structure and dynamics of the RNA duplexes with central Ψ-A base pairs in a systematic way, we carried out 500 ns MD simulations under the same conditions for all the duplexes specified in Table 1. The analysis of the MD trajectories aimed to answer the following questions: (i) for a given sequence, how does the presence of Ψ influence the conformation and flexibility of the modified duplex compared with its unmodified counterpart, (ii) whether the impact of Ψ depends on the identities of the adjacent base pairs. The analysis of the trajectories showed that both the modified and unmodified duplexes retained the initial A-form geometry with root-mean-square deviations (RMSDs) of approximately 1.4 Å (Supplementary Table S7 and Supplementary Fig. S1).

To probe the influence of the Ψ modification on flexibility of the RNA duplexes, we determined the root-mean-square fluctuations (RMSFs) per residue for the backbone atoms (P, OP1, OP2, O5′, C5′ and O3′) and the order parameters (*S*^2^) for sugar C1′-H1′ vectors. We found that in the duplexes with Ψ modification, the backbone flexibility was only marginally lower than that in the corresponding reference duplexes. For the duplexes with the central RΨY motif (R-purine and Y-pyrimidine), the decrease in RMSF was mainly localized in residues 5 and 6, whereas for the YΨR motif, this effect propagated along the entire strand (Supplementary Fig. S2). The *S*^2^ was higher than 0.87 when excluding terminal residues, that indicates low flexibility for the nucleotides within the duplexes, both with and without Ψ (Supplementary Fig. S3). The *S*^2^ was only marginally higher for residues in the modified strand in duplexes with YΨR motif.

**Figure 4:**
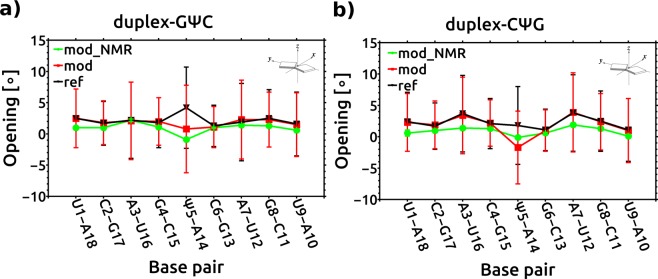
Average opening values (deg) for the base pairs in (**a**) duplex-GΨC and (**b**) duplex CΨG. Green – Ψ-modified NMR models; Red – Ψ-modified duplexes from MD simulation; Black – reference, unmodified from MD simulation. Vertical lines represent standard deviations.

To gain insight into the structural differences between the RNA duplexes with central Ψ-A base pairs and their unmodified counterparts at a more detailed and local level, we calculated the helical parameters^34^ (Fig. 4, Supplementary Table S8, Supplementary Figs S4 and S5). Overall, the replacement of the base pair U-A by Ψ-A induced only slight changes in certain base pair and base pair step parameters. For example, for the Ψ5-A14 base pairs, we observed slightly smaller values (by 3–4°) for the base pair openings compared with those obtained for the U5-A14 base pairs (Fig. 4 and Supplementary Fig. S4). The Ψ-modified duplexes displayed similar roll values at each base pair step to those of their unmodified counterparts (Supplementary Fig. S5b). Our results agree with the previously reported sequence dependence for the roll values of RNA double helices [pyrimidine-purine (YR) > purine-purine (RR) > purine-pyrimidine (RY)]^36^. We observed small increments in the backbone P4-P5 distances and decrements in the P3-P4 and P5-P6 distances in all the Ψ-modified strands (Supplementary Fig. S6). Further, we noticed small increments (~0.2 Å) in the interstrand C1′-C1′ distances for the Ψ5-A14 base pairs compared with those of the U5-A14 base pairs (Supplementary Fig. S7). The glycosidic torsion angle (χ) for the Ψ5 was shifted 10° towards lower *anti* values (~195°) compared with that of U5 (~205°) in all the duplexes studied (Supplementary Fig. S8). In summary, we conclude that all the duplexes with central Ψ-A base-pairs maintain A-form conformations. The Ψ modification only subtly alters the local structure with marginal dependence on the sequence, which implies similar base stacking geometries in the modified and unmodified duplexes.

### Additional water bridge in the Ψ-modified duplexes

**Figure 5:**
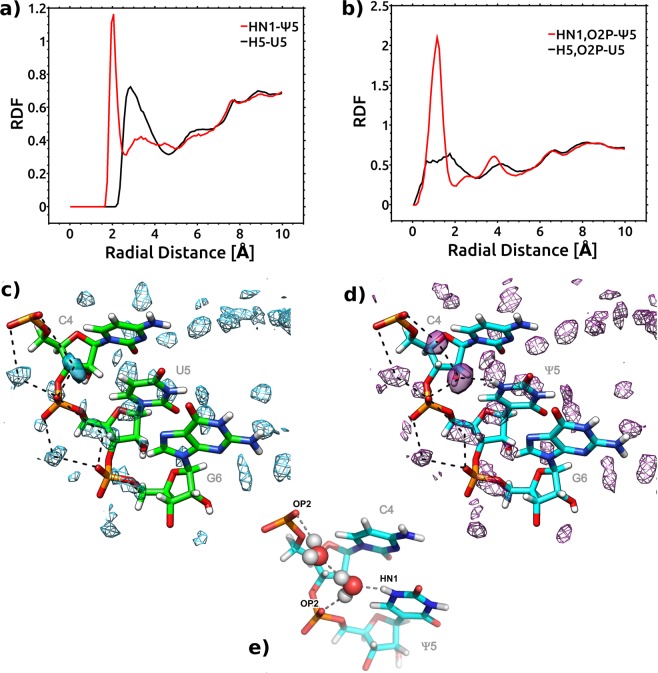
Change in the hydration pattern upon U to Ψ modification for duplex-CΨG and duplex-CUG. RDFs of water oxygen atoms (**a**) around the HN1-Ψ5 and H5-U5 atoms, respectively; (**b**) around the geometric center of the OP2-Ψ5 and HN1-Ψ5 atoms or OP2-U5 and H5-U5 atoms. Red – Ψ-modified duplexes; Black – unmodified duplexes. Water occupancy contoured at equivalent levels and hydrogen bonding patterns (in dotted lines) viewed from the major groove. (**c**) Reference duplex-CUG; (**d**) Ψ-modified duplex-CΨG; differences in hydration patterns are shown in solid. (**e**) Snapshot of two water molecules making contact between HN1, OP2 atoms of Ψ and OP2 atom of the preceding residue.

MD simulations in explicit solvents are well suited to analyze hydration, especially regarding predictions of highly specific long-residency hydration sites^37^. To identify differences in the hydration patterns of Ψ-modified and unmodified duplexes, we calculated the radial distribution functions (RDFs) around the selected atoms in the modification region. The RDF plot of the water oxygen atoms with respect to the HN1-Ψ5 atom (Fig. 5a) for the duplex-CΨG indicates the formation of a well-defined first hydration shell between 1.5 Å and 2.5 Å with a maximum at 2.05 Å. For the corresponding hydrogen atom H5 in the unmodified U5 base, the water molecules are farther from the base. The first solvation peak of H5-U5 corresponds to the second peak around atom HN1-Ψ5, but fewer water molecules were found at this distance in the modified duplex. The distribution of water oxygens between the base and the backbone was found to be highly ordered around the geometric center between the OP2 atom and the HN1 atom of the Ψ5 residue (Fig. 5b). In general, the RDFs were not very sensitive to the sequence context of the RNA duplexes.

To compare the structure of the water around the Ψ and the corresponding U in modified and unmodified duplexes at the atomic level, we calculated the occupancy of the water-mediated hydrogen bonding bridges. In the simulations of the Ψ-modified duplexes, a frequent intramolecular water bridge was formed between the HN1 and OP2 atoms of Ψ5, with an occupancy of 28–29% (Table 3). This configuration was the most frequent water bridge seen around the duplexes, with the occupancy slightly higher than that of the water bridges between the adjacent phosphate oxygens (up to 24%). The change in the hydration pattern due to the Ψ modification also manifested as an increase in the percent of bridging water between the adjacent phosphate oxygen atoms of residues 5 and 6 and a decrease in that between residues 4 and 5. The hydrogen bond between water oxygen and HN1-Ψ5 atom displayed a short lifetime of 0.02 ns in comparison to the long lifetime for the waters hydrogen-bonded to the OP1 and OP2 atoms (0.5–0.7 ns). In modified duplexes the hydrogen-bonding lifetime to the O2P-Ψ5 and O2P of preceding residue increased to 1–1.1 ns.

**Table 3:** Water bridges (occupancy in %) formed by the backbone atoms near the modified residue and its unmodified counterpart.

Water bridge	duplex-GΨC		duplex-CΨG		duplex-AΨU		duplex-UΨA	
	mod	ref	mod	ref	mod	ref	mod	ref
OP2(5)-W-HN1(5)	28	–	29	–	28	–	28	–
OP2(5)-W-OP1(4)	12	16	5	10	10	14	7	13
OP2(5)-W-OP2(4)	7	11	<5	9	6	9	8	13
OP2(5)-W-O5′(4)	<5	6	<5	5	<5	5	<5	<5
OP2(6)-W-OP1(5)	21	15	22	10	20	15	17	9
OP2(6)-W-OP2(5)	16	12	24	18	19	16	19	13
OP2(6)-W-O5′(5)	<5	<5	<5	<5	5	<5	<5	<5

Abbreviations ‘mod’ and ‘ref’ stand for the modified and reference, unmodified duplexes, respectively.

To visualize the differences in the hydration patterns around the residues Ψ5 and U5 within the duplexes, the water molecules over the MD trajectories were mapped. Figure 5d shows an additional hydration pocket around the HN1-Ψ5 hydrogen in the major groove, other than the phosphate backbone bridging water. This additional hydration site was identified in all the Ψ-modified duplexes and was not present in the unmodified reference structures (Fig. 5c). In our simulations, a water molecule bridges the HN1 and OP2 atoms within the same Ψ residue, but the water molecule is not directly bonded to the OP2 atom of the preceding residue. On the other hand, this bridging water molecule is a proton donor to another water that is coordinated with the 5′-neighboring phosphate. As a result, the HN1-Ψ5 is connected with the 5′-neighboring OP2 atom with a chain of two water molecules (Fig. 5e). These observations were similar for all the RNA duplexes studied.

### Stacking Interactions

**Figure 6:**
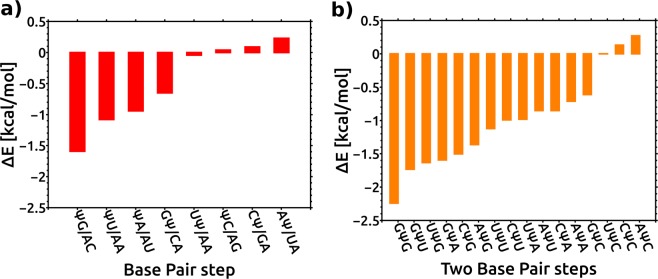
Impact of Ψ on the intrinsic stacking energies. (**a**) Change in the QM stacking energy between base pairs at a given base pair step upon Ψ modification (ΔE = E_modif_ − E_unmodif_). ΨG/AC denotes the 5′ΨG/3′AC base pair step and so on. (**b**) Prediction of the impact of Ψ on the stacking energies at trinucleotide steps based on QM calculations. GΨG denotes the 5′GΨG/3′CAC motif and so on. The base pair stacking energies at trinucleotide steps were calculated by the sum of the base pair stacking energies for two consecutive dinucleotide steps. The data are ordered in decreasing stability of eight unique dinucleotide steps/ sixteen unique trinucleotide steps in RNA duplexes.

Our structural analysis revealed minimal effects on the base stacking geometry within the RNA duplexes from Ψ modification in different sequence contexts. However, the Ψ modification changes the electron cloud (charge distribution) compared with unmodified U and thus could influence the base stacking at base pair steps in the duplex^38^. To further explore the effect of Ψ on the stacking interactions, we used high level quantum mechanical (QM) method to study stacking interaction energies for eight base pair steps containing the Ψ-A and the reference U-A base pairs. The calculations were performed using the geometries of the stacked base pairs derived from explicit solvent MD simulations of the respective RNA duplexes (Supplementary Fig. S9, Supplementary Methods, Section 6). Figure 6a summarizes the changes in the stacking energies at each base pair step when U was replaced with Ψ. In addition, the base pair step stacking energies for eight unique base pair steps containing the Ψ-A base pair and their unmodified counterparts are shown in Supplementary Table S9 and Supplementary Fig. S10. These results show the clear dependence of the change in the stacking energies on the sequence context, ranging from −1.59 to 0.23 kcal/mol. The base pair steps can be divided into two groups. In the first group, Ψ stabilizes the base pair step stacking energies, whereas in the second group, no stabilization is observed. The Ψ stabilized the base pair step stacking interactions when it was 5′ to G, U and A (5′ΨG/3′AC, 5′ΨU/3′AA and 5′ΨA/3′UU base pair steps) and when Ψ was 3′ to G (5′GΨ/3′CA base pair step). In contrast, the Ψ at the 3′ position to A, C, and U (5′AΨ/3′UA, 5′CΨ/3′GU and 5′UΨ/3′AA base pair steps) did not stabilize the base pair step stacking interactions. The stabilization effect was also not observed when Ψ was at 5′ to C (5′ΨC/3′AG base pair step). The largest stabilization effect, −1.59 kcal/mol, was observed at the 5′ΨG/3′AC base pair step. In summary, the Ψ stabilized the stacking energies at the ΨR base pair steps, whereas at the YΨ base pair steps, no stabilization was observed. At the RΨ and ΨY base pair steps, the stabilization effect depended on the R and Y base identity. The analysis of the decomposition of the stacking energies into intra- and interstrand terms showed that their contributions depended on the sequence (Supplementary Table S9 and Supplementary Fig. S11). For comparison, we evaluated the stacking energies of the base pair steps using empirical molecular mechanics force field (ΔE_MM_). Our MM-based calculations were performed over the trajectories, thus assessing the dynamic nature of the systems. On the average, the variability of the stacking energy for the base pair steps along the RNA trajectory was approximately 2.2 kcal/mol. For four base pair steps (5′ΨG/3′AC, 5′ΨU/3′AA, 5′GΨ/3′CA and 5′ΨA/3′AU) where the calculations at the QM level (ΔE_QM_) showed stabilization in the stacking interaction energies from the Ψ modification, the MM-based calculations (ΔE_MM_) displayed the same stabilization order (Supplementary Fig. S13). For the remaining four base pair steps, although the results at the QM level and from MM-based calculations showed some discrepancies, both the ΔE_MM_ and ΔE_QM_ revealed that, at these base pair steps, replacement of U with Ψ change the stacking energies less than at the base pair steps from the first group. When only the contribution of the Ψ to the intrastrand stacking energy between the bases in a single strand of RNA duplex was considered, we obtained the same ordering both for the QM-based and the MM-based ΔEs (Supplementary Fig. S14). This result agrees with previous calculations that showed that QM gas phase stacking energies are consistent with MM-based calculations obtained with an AMBER force field^38–40^.

Based on the changes in the stacking energies upon replacing U with Ψ calculated for the eight unique base pair steps, it was encouraging to predict the impact of Ψ on the stacking energy within the trinucleotide motifs with Ψ-A at the central position when the U-A base pairs were replaced with Ψ-A. There are 16 unique trinucleotide motifs with central Ψ-A base pairs (Fig. 6b). The predictions revealed sequence-dependent variations of the Ψ contribution to the base stacking energies. The strongest stabilization from Ψ modification is predicted for the 5′GΨG/3′CAC motif by both the QM-based and MM-based calculations (Supplementary Fig. S15). Both calculation methods predicted no stabilization for the stacking interactions after replacing U with Ψ for the 5′UΨC/3′AAG and 5′CΨC/3′GAG motifs. Our results showed that the effect of pseudouridine stabilization can differ by up to approximately 2.5 kcal/mol depending on the sequence context; however, for several sequence motifs, the stabilization effect is in the range of approximately −1.5 to  −0.5 kcal/mol. The change in the stacking energies upon replacing U with Ψ to obtain an internal Ψ-A base pair at the GΨC and CΨG steps correlates with the experimental result^31^ showing a more favorable thermodynamic effect for Ψ modification of duplex-CΨG than that of duplex-GΨC (Supplementary Fig. S16). To visualize the sequence dependent impact of Ψ-A base pairs on the interactions with the adjacent base pairs within the RNA duplex, we generated isodensity surfaces color-coded by electrostatic potential (Supplementary Fig. S17).

In summary, analysis of our calculations suggests that the intrinsic base stacking is the main factor that contributes to the sequence dependence of the thermodynamic stabilities of the RNA duplexes due to the introduction of Ψ modifications.

## Discussion

Currently, the proposed mechanisms for the stabilization effect of Ψ include structural water coordinated by HN1-Ψ, improved stacking properties and the preference for the C3′-*endo* sugar pucker^12^. A structural water molecule coordinated with the HN1 of the Ψ was previously seen in the electron density maps of tRNA for residues Ψ38 and Ψ39 at the anticodon loop^29^ and most recently, for example, in the crystal structures of tRNA (PDB ID: 1EHZ), rRNA and in U2 snRNA-branch point duplexes (PDB ID: 3CGP, 3CGR, 3CGS). Different scenarios of interactions mediated by the pseudouridine-bound water molecule were observed: a single water molecule bridging the HN1, OP2 atoms of the Ψ and OP2 of the preceding residue^29^ (PDB ID: 1EHZ, 3CGR, 3CGS), an intra-residue bridge only (Ψ2621 in PDB ID: 1S72) or two water molecules between the HN1 and OP2 atoms of the Ψ and the OP2 of the preceding residue (PDB ID: 3CGP). Ψ intra-residue water bridge was present in all of the above structures whereas inter-residue water bridge to the OP2 atom of the 5′ residue was formed at the loop or stem/loop interface structural context. In our duplexes, both from the NMR structures and from the MD trajectories, the distance between the HN1 atom of the Ψ and the OP2 atom of the 5′ residue was too large to make a single water bridge (6.3 Å and 6.5 Å for the NMR structures of the duplex-GΨC and duplex-CΨG, respectively and the peak at 6.75 Å from the distance distributions in the MD simulations of all the Ψ-modified duplexes, Supplemental Fig. S18). Distances smaller than 5.4 Å (as present in the PDB ID: 3CGR, 3CGS structures with single water molecules) were observed for 3–4% of the conformations with the YΨ motif and 8% of the conformations with the RΨ. In our MD simulations of the Ψ-modified duplexes, we observed a water bridge formed by two water molecules. No long-lived hydrogen bonds involving the HN1-Ψ5 atom were observed. Distances similar to those in our duplexes were measured between the HN1 atom of Ψ and the OP2 atom of the preceding residue in other NMR structures with Ψ embedded in a double helix (6.3 Å and 6.4 Å in the duplex PDB ID: 5OR0 and 5.1–6.9 Å in the duplex PDB ID: 2KYE, depending on the model). The presence of long-lived water bridges between the OP2 atoms of consecutive residues and the HN1 atom of Ψ32 was previously reported from a 500 ps MD simulation of the tRNA^Asp^ hairpin performed with SPC/E water and NH4^+^ with the AMBER FF94 force field by Auffinger and Westhof^41,42^. The two bridging water molecules seen in our simulations may be characteristic of the TIP3P water model. It has been reported that the choice of water model has a visible impact on the predicted structure and structural dynamics of RNA^37,43^.

We conclude that internal single water bridges between the HN1 and OP2 atoms of Ψ likely characterize Ψ in all structural contexts. In loops and at stem-loop junctions, this water also could form a hydrogen bond with the OP2 atom of the preceding residue and make a long-lived water bridge. In the context of a regular helix, the water molecule forms an internal bridge within Ψ, but instead of directly binding to the OP2 atom of the preceding residue, more often is hydrogen bonded by an additional water molecule, making a string of two water molecules.

The effect of Ψ associated with its base stacking properties has so far not been systematically investigated, and our work emphasizes this aspect of the Ψ contribution to the RNA duplex stability. We calculated intrinsic stacking energies at different base pair steps using the QM method and structures derived from MD simulations. The choice of the representative geometry is discussed in the Section 6 of the Supplementary Methods. Our results showed that the changes in the base stacking energies from Ψ modification depended on the sequence; in most of the sequence contexts, the Ψ stabilized the stacking interactions but not always. Although these calculations cannot be directly used for the prediction of the relative thermodynamics of duplex formation^44^, the calculations suggest that the stacking interactions are the key factor in the sequence context dependence of the enhanced stability of the RNA with Ψ. The duplex formation could be conceptually considered to be a two-step process: (i) the transition of single strands from an unstacked to a stacked, ordered state and (ii) the association of two preorganized single-stranded helices to form a duplex^45,46^. The preference for the C3′*-endo* sugar pucker conformation of Ψ appears to play a more important role in the process (i). The stabilization of the C3′*-endo* sugar pucker conformation in single stranded RNAs has been demonstrated experimentally^12^. In addition, stacking interactions and structural water likely contribute to the enhanced preorganization of single stranded RNA with pseudouridine. The contribution of Ψ to single strand preorganization has recently been supported by experimental data and MD studies^47^. In effect, Ψ modification resulted in preorganization of single strands with nucleotides in the C3′-*endo*, *anti* and axial conformations, thereby decreasing the entropic cost of duplex formation and increasing the thermodynamic stability of the duplex. The modifications that operate by the mechanism of reducing unfavorable entropy when going from a single-stranded to a double-stranded molecule include 2′-O-methyl-modified residues, LNA nucleosides^48^ and 2-thiouridine^46^. In process (ii), i.e., helix association, Ψ does not play significant role because it does not change the hydrogen bonding, and the structural water molecule is not at the binding interface of the two strands. To test if the differences in the thermodynamics of the duplex formation of the Ψ-modified and unmodified duplexes can be obtained by computational predictions, we employed the MM-PBSA method. The relative binding free energies calculated for the Ψ-modified and unmodified duplexes were between 0.61 to −1.47 kcal/mol and did not follow the experimental trend (Supplemental Table S10). However, the MM-PBSA calculations correspond to process (ii) and assume that the separated single strands retained the same A-form structure as assumed by the duplex and thus do not exactly mimic the experimental measurements. The single strands are presumably more flexible than double helices. Unfortunately, the calculations of the entropic and enthalpic contributions from the single strand preorganization to the duplex formation, i.e., process (i), are beyond the scope of MM-PBSA method with the single trajectory approach (see Supplementary Methods, Section 5). The results from the MM-PBSA calculations indirectly indicated that process (i) rather than (ii) is responsible for the sequence dependent stabilization effect of Ψ. The MM-PBSA method has been previously used for predicting the thermodynamics of duplex formation for RNA duplexes with modifications that alter hydrogen bond pairing^48–50^. From our QM and MM calculations (Supplemental Table S9), Ψ slightly destabilized the Ψ-A base pair interactions in agreement with previous work^51,52^. Once the duplex is formed, all nucleotides have the C3′-*endo* conformation, and we have not seen any additional ordering of the ribose moiety of Ψ compared with that of U.

In the future, it would be interesting to evaluate the effect of Ψ on single strand conformation in different sequence contexts with both experimental and computational methods.

## Methods

### RNA synthesis

The RNA oligonucleotides (5′-UCAGΨCAGU-3′, 5′-ACUGACUGA-3′, 5′-UCACΨGAGU-3′, 5′-ACUCAGUGA-3′) were synthesized on an Applied Biosystems DNA/RNA synthesizer, deprotected and purified according to previously published procedures^31,53,54^. For NMR study, each of the obtained duplexes was dissolved in a volume of 200 μl of 90% H_2_O and 10% D_2_O solution and placed into a 3 mm NMR sample tube (Supplementary Methods, Section 1).

### NMR measurements

The NMR spectra were acquired on a Bruker Avance III 700 MHz spectrometer, equipped with a QCI CryoProbe. The two-dimensional NOESY experiments involving non-exchangeable ^1^H resonances were recorded at 25 °C. To facilitate peak assignments and obtain additional structural information 2D NOESY spectra at 30 and 35 °C were also acquired for duplex-GΨC and -CΨG, respectively, with the broad spectral width of ~7002 Hz. High resolution DQF-COSY spectra were acquired with narrow spectral width of 2520 Hz. Natural abundance ^1^H-^13^C HSQC experiments (25 °C) were performed within the spectral width of 7002 Hz in the ^1^H dimension and 28248 Hz in the ^13^C dimension. To increase the resolution ^1^H-^13^C HSQC spectra were acquired with narrowed spectral range of 4901 Hz in ^1^H dimension and 10570 Hz in ^13^C dimension and 64 scans per t1 increment. Proton detected ^1^H-^31^P COSY spectra were recorded at 25 °C within 2102 Hz spectral range in the ^1^H dimension and 1417 Hz in the ^31^P dimension. To aid ^1^H resonance assignment a two-dimensional ^1^H-^31^P hetero TOCSY-NOESY experiments were recorded at 25 °C with a DIPSI-2 sequence^55^ using 20 ms spin-lock period and 500 ms NOESY mixing time. The spectra were acquired with a spectral width of 10504 Hz in ^1^H and 1701 Hz in the ^31^P dimension. Exchangeable ^1^H resonances were analyzed using the 2D NOESY spectra obtained at 15 °C with a 100 ms mixing time. The residual water peak for homonuclear experiments in D_2_O was suppressed using low-power presaturation whereas solvent suppression for samples in 90% H_2_O/10% D_2_O was achieved by applying a pulse sequence using excitation sculpting with gradients^56^. A sodium 2,2-dimethyl-2-silapentane-5-sulfonic acid (DSS) was used as an internal chemical shift reference. Collected spectra were processed with TopSpin (Bruker, Inc.) and analyzed with Felix (Felix NMR, Inc.) software.

### Structural restraints

NOE restraints for non-exchangeable protons were obtained from analysis of 2D NOESY spectra (D_2_O) with mixing times of 100 and 150 ms recorded at 25 °C using the Isolated Spin Pair Approximation (ISPA) approach. An average pyridine H5-H6 peak volumes were used as a reference with a distance of 2.45 Å. Error bounds for the restraints were set to −15% (lower) and +30% (upper) of the calculated interproton distance. The details for the identification of Watson-Crick base pairs and hydrogen bonding pattern between Ψ and A bases, constraints for endocyclic and backbone torsion angles α, β, γ, ε and ζ, sugar pucker conformations and orientations of the nucleobases with respect to the sugar rings are summarized in Supplementary Methods, Section 2.

### Computational details

All molecular mechanics (MM) calculations and molecular dynamic (MD) simulations were performed with AMBER 14 simulation package and AmberTools15^57^. All MD simulations were carried out with CPU and GPU version of *pmemd* module using ff99bsc0χ_OL3_ force field^58–60^ for regular RNA and parameter set for Ψ reported by Deb *et al*.^61^. The 9-bp RNA duplexes were built as regular RNA duplexes in A-RNA conformation with Nucleic Acid Builder (NAB). The Ψ modification was incorporated at the 5^th^ position of the initial structures by rearranging the atom positions of the uridine residue.

### Structure calculations by simulated annealing method

The structure calculations for the 9-bp Ψ-modified RNA duplexes (duplex-GΨC and -CΨG) were carried out with the starting coordinates in the A-RNA conformation by the simulated annealing method^62^. During the simulated annealing calculations, restrained MD simulations were performed using NOE structural restraints collected from the analysis of NMR specta (see Supplementary Materials and Methods, Section 2). A generalized Born implicit solvent model was used with a salt concentration of 150 mM NaCl.

The energy minimized structure of each RNA duplex was subjected to two cycles of a simulated annealing procedure following Chen *et al*.^63^ (see Supplementary Methods, Section 3). The 100 structures generated after the first cycle of simulated annealing runs were sorted based on the violations criteria of 0.0 kcal.mol^−1^ penalty in torsional restraints and minimum penalty (~1.67 kcal.mol^−1^ for duplex-GΨC and ~0.15 kcal.mol^−1^ for duplex-CΨG) for distance restraints. Among these sorted structures, 20 structures, without any restraint violations higher than 0.1 Å and with minimum number of restraint violations less than 0.1 Å, were selected for the next cycle of simulated annealing run. In the second cycle, these 20 structures were further refined to extract an ensemble of 10 final structures applying the similar violation criteria (~1.39 kcal.mol^−1^ for duplex-GΨC and ~0.17 kcal.mol^−1^ for duplex-CΨG) chosen in the first cycle.

### Molecular dynamics (md) simulations

Each of the structures was solvated with TIP3P^64^ water molecules in truncated octahedral boxes with minimal distance of 10 Å from the solute border and periodic boundary conditions were applied. The systems were neutralized with Na^+^ ions and KCl excess salt was added to obtain the concentration of 1.0 M by using Joung and Cheatham ion parameters^65^ to resemble conditions of UV melting experiments. The solvated systems were minimized and equilibrated. For each system, during the production run constant pressure Langevin dynamics was performed at 300 K for a total 500 ns with a 2 fs time step and trajectory files were written at each 10 ps. The pressure was regulated using Berendsen barostat^66^ at reference pressure of 1 atm. Electrostatics was handled with PME^67^ method with a direct space cutoff of 10.0 Å (see Supplementary Methods, Section 4).

### Analysis of MD trajectories

The utilities available with the *cpptraj* module of AmberTools 15^68^ were used for the trajectory analysis. The water occupancy maps around the average MD structure were calculated using the *grid* routine and visualized using UCSF-Chimera^69^. Hydrogen bond formations were considered if (i) the donor-acceptor distance was ≤3.0 Å, and (ii) the donor-hydrogen-acceptor angle was ≥135.0°. The *lie* utility in the *cpptraj* program was used for the calculation of the molecular mechanics (MM) force field-based base stacking energies as a summation of pairwise electrostatic and van der Waals interaction energies between the base atoms only. Cluster analysis was performed with *cpptraj* using the average-linkage clustering algorithm (for details see Supplementary Methods, Section 5). The order parameters (*S*^2^) were calculated for C1′-H1′ vector using the *ired* utility in the *cpptraj* program (for details see Supplementary Methods, Section 5). Structural parameters were calculated using Curves+/Canal programs^34^.

### *Ab initio* base pair step stacking energies

The *ab initio* base pair step stacking energies were calculated in the gas phase with a density functional theory (DFT) approach at the B97D/Def2TZVPP level of theory using the GAUSSIAN09 software suite. The intra- and interstrand base stacking energies were calculated following Svozil *et al*.^70^ (for details see Supplementary Methods, Section 6). The QM base pair step stacking energies were calculated by subtracting the hydrogen bonding interaction energies from the interaction energies of the complete four-base stacked systems (Supplementary Fig. S19) according to the equation: Δ[E]_AC,BD_ = [E]_ADBC_ − ([E]_AD_ + [E]_BC_), where [E]_ADBC_ represents the interaction energy of the complete four-base stacked system, and [E]_AD_ and [E]_BC_ represent hydrogen bonding interaction energies in the Watson-Crick base pairs. The interaction energies were calculated by subtracting the monomer total energies from the corresponding dimer/tetramer energies corrected for basis set superposition error (BSSE) via the counterpoise method implemented in the GAUSSIAN09 software suite. The intrastrand ([E]_AB_ and [E]_CD_) and interstrand ([E]_AC_ and [E]_BD_) interaction energies were also reported in Supplementary Table S9. Stacking energies between bases in a single strand of RNA duplex at given base step represented by the intrastrand energies are shown on Supplementary Fig. S14. The geometries of the base pair steps were derived from the average coordinates of the most populated cluster obtained from the cluster analysis (Supplementary Methods, Section 6). The four-base stacked geometries for QM calculations were prepared by superposition of previously optimized monomers of individual bases on the scaffold of the average structures.

## Supplementary information

Supplementary information

## Data Availability

Coordinates have been deposited in the Protein Data Bank (PDB IDs: 6I1W and 6I1V for the duplex-GΨC and duplex-CΨG, respectively) and the NMR data in the Biological Magnetic Resonance Data Bank (BMRB codes: 34324 and 34323 for the duplex-GΨC and duplex-CΨG, respectively).
