# Supplementary material for: Computational and NMR studies of RNA duplexes with an internal pseudouridine-adenosine base pair
Source: Sci Rep. 2019 Nov 7;9:16278. doi: 10.1038/s41598-019-52637-0 (PMC6838189; doi:10.1038/s41598-019-52637-0)
Supplement: Supplementary file 1 — Supplementary information [file 41598_2019_52637_MOESM1_ESM.pdf]

Supplementary Information

Computational and NMR studies of RNA duplexes with an internal pseudouridine-adenosine base pair

Indrajit Deb[1,2,+], Łukasz Popenda[3,+], Joanna Sarzyńska[1,*], Magdalena Małgowska[1], Ansuman Lahiri[2], Zofia Gdaniec[1] and Ryszard Kierzek[1]

[1] Institute of Bioorganic Chemistry, Polish Academy of Sciences, Noskowskiego 12/14, 61-704 Poznan, Poland
[2] Department of Biophysics, Molecular Biology & Bioinformatics, University of Calcutta, Kolkata 700009, West Bengal, India
[3] NanoBioMedical Centre, Adam Mickiewicz University, Umultowska 85, 61-614 Poznan, Poland

[+] these authors contributed equally to this work

* Corresponding author
Joanna Sarzynska
Institute of Bioorganic Chemistry
Polish Academy of Sciences
Noskowskiego 12/14
61-704 Poznan, Poland
e-mail: joanna.sarzynska@ibch.poznan.pl

Supplementary Tables………… 2
Supplementary Figures………… 14
Supplementary Methods ………… 36
Supplementary References ………… 40

SUPPLEMENTARY TABLES

Supplementary Table S1. ^{1}H chemical shifts of the duplex-GΨC recorded in D_2O at 25 °C.

Res.	H6 / H8	H2 / H5	H1′	H2′	H3′	H4′	H5′ / H5″	amino[1]	imino[1]
**U1**	8.10	5.85	5.57	4.57	4.54	4.33	4.03 / 3.91	–	
**C2**	8.05	5.86	5.68	4.60	4.68	4.51	4.55 / 4.22	8.42 / 7.04	–
**A3**	8.09	7.04	5.94	4.73	4.74	4.55	4.59 / 4.24		–
**G4**	7.26	–	5.60	4.52	4.52	4.47	4.51 / 4.08		13.41
**Ψ5**	7.16	–	4.74	4.41	4.46	4.23	4.45 / 4.02	–	10.26 / 14.16
**C6**	7.85	5.65	5.53	4.53	4.57	4.45	4.55 / 4.11	8.32 / 6.93	–
**A7**	8.02	7.03	5.87	4.68	4.71	4.52	4.58 / 4.17		–
**G8**	7.19	–	5.58	4.31	4.40	4.42	4.49 / 4.06		13.56
**U9**	7.58	5.15	5.77	4.07	4.15	4.20	4.43 / 4.04	–	
**A10**	8.38	8.01	5.80	4.79	4.57	4.35	4.06 / 3.93		–
**C11**	7.70	5.29	5.49	4.28	4.47	4.49	4.54 / 4.16	8.32 / 6.90	–
**U12**	7.89	5.41	5.58	4.63	4.65	4.45	4.57 / 4.15	–	13.52
**G13**	7.72	–	5.75	4.58	4.68	4.53	4.53 / 4.21		11.75
**A14**	7.94	7.72	5.92	4.55	4.62	4.51	4.61 / 4.17		–
**C15**	7.41	5.17	5.33	4.22	4.34	4.40	4.50 / 4.04	8.30 / 6.96	–
**U16**	7.82	5.34	5.53	4.60	4.60	4.41	4.55 / 4.09	–	13.43
**G17**	7.67	–	5.73	4.45	4.61	4.49	4.51 / 4.15		11.92
**A18**	7.85	7.88	6.00	4.09	4.30	4.28	4.53 / 4.08		–

[1] Exchangeable ^{1}H chemical shifts are determined in 90% H_2O / 10% D_2O at 15 °C.

Supplementary Table S2. ^{13}C and ^{31}P chemical shifts of the duplex-GΨC recorded in D_2O at 25 °C.

Res.	C6 / C8	C2 / C5	C1′	C2′	C3′	C4′	C5′	^{31}P
**U1**	143.7	104.1	93.4		73.7		61.7	–
**C2**	142.0	98.1	93.8		72.2			-4.00
**A3**	139.6	152.5	92.8	75.6	72.9	82.0		-3.71
**G4**	136.2	–	92.6	75.4				-3.86
**Ψ5**	140.1	–	82.8	74.8		79.7	65.2	-3.91
**C6**		97.9	93.8		72.4			-3.85
**A7**	139.5	152.5	92.9	75.6				-3.81
**G8**	135.9	–	92.7	75.5	72.8			-3.93
**U9**	141.8	103.4	92.3	77.3	70.1	84.0		-4.31
**A10**	141.4	154.1		75.2			61.7	–
**C11**	141.3	97.0	93.5	75.6	72.2		64.7	-4.27
**U12**		103.6	93.4	75.3	72.4			-4.23
**G13**	136.4	–	92.3	75.4				-3.73
**A14**	139.3	154.0	92.8	75.6	72.7		65.0	-4.10
**C15**	140.6	97.2	93.9	75.5	72.3			-4.20
**U16**		103.6	93.5	75.2	72.4			-4.33
**G17**	136.4	–	92.7	75.5	73.0		65.6	-3.81
**A18**	140.1	154.6	91.7	77.9	70.2	83.9		-4.04

Supplementary Table S3. ^{1}H chemical shifts of the duplex-CΨG recorded in D_2O at 25 °C.

Res.	H6 / H8	H2 / H5	H1′	H2′	H3′	H4′	H5′ / H5″	amino*	imino*
**U1**	8.13	5.87	5.59	4.58	4.55	4.35	4.05 / 3.92	–	
**C2**	8.08	5.89	5.69	4.56	4.69	4.52	4.56 / 4.23	8.50 / 7.10	–
**A3**	8.16	7.38	5.95	4.55	4.74	4.53	4.63 / 4.22		–
**C4**	7.56	5.24	5.43	4.37	4.44	4.42	4.54 / 4.08	8.40 / 6.96	–
**Ψ5**	7.32	–	4.75	4.50	4.56	4.24	4.42 / 4.04	–	10.45 / 13.36
**G6**	7.61	–	5.71	4.58	4.67	4.53	4.54 / 4.12		11.58
**A7**	7.76	7.45	5.88	4.65	4.65	4.50	4.58 / 4.14		–
**G8**	7.12	–	5.59	4.32	4.37	4.42	4.47 / 4.03		13.60
**U9**	7.59	5.17	5.79	4.07	4.16	4.21	4.44 / 4.04	–	
**A10**	8.42	8.06	5.85	4.81	4.60	4.38	4.08 / 3.95		–
**C11**	7.75	5.33	5.50	4.30	4.50	4.49	4.55 / 4.18	8.40 / 6.96	–
**U12**	7.98	5.42	5.59	4.53	4.59	4.47	4.58 / 4.15	–	14.22
**C13**	7.90	5.71	5.58	4.53	4.60	4.48	4.58 / 4.16	8.22 / 6.93	–
**A14**	8.02	6.90	5.88	4.69	4.70	4.53	4.57 / 4.18		–
**G15**	7.13	–	5.56	4.40	4.41	4.46	4.49 / 4.06		13.20
**U16**	7.70	5.01	5.53	4.59	4.55	4.41	4.55 / 4.09	–	13.68
**G17**	7.63	–	5.75	4.46	4.59	4.49	4.49 / 4.14		12.01
**A18**	7.86	7.91	6.01	4.09	4.31	4.29	4.54 / 4.09		–

* Exchangeable ^{1}H chemical shifts are determined in 90% H_2O / 10% D_2O at 15 °C.

Supplementary Table S4. ^{13}C and ^{31}P chemical shifts of the duplex-CΨG recorded in D_2O at 25 °C.

Res.	C6 / C8	C2 / C5	C1′	C2′	C3′	C4′	C5′	^{31}P
**U1**	143.8	104.2	93.5		73.9		61.9	–
**C2**	142.1	98.2	93.8		72.8			-3.91
**A3**	139.6	153.3	92.9	75.6	72.7	82.0	65.1	-3.85
**C4**	140.9	97.0	93.4	75.5				-4.04
**Ψ5**	140.4	–	83.0	74.9		79.6	65.5	-3.69
**G6**	136.4	−	92.7		72.8			-3.61
**A7**	139.2	153.3	92.8	75.6	72.7			-3.85
**G8**	135.8	–	92.7	75.6	72.9			-3.90
**U9**	141.9	103.5	92.2	77.3	70.3	84.2	65.5	-4.31
**A10**	141.4	154.3	92.9	75.3	74.0		62.0	–
**C11**	141.3	97.2	94.1	75.6	72.3		64.8	-4.15
**U12**	142.3	103.1	93.7	75.3				-4.36
**C13**	143.7	97.9	93.8	75.4				-3.98
**A14**	139.4	152.3	92.8	75.7	73.0		65.3	-3.77
**G15**	135.9	−	93.0	75.1	72.8			-3.91
**U16**	141.0	102.9	93.4	75.3	72.2			-4.50
**G17**	136.3	–	92.7	75.6	73.1		65.7	-3.73
**A18**	140.0	154.7	91.6	77.9	70.3	83.9		-4.01

Supplementary Table S5. Local base-pair parameters in solution NMR structures for (A) duplex-GΨC and (B) duplex-CΨG averaged over 10 models (standard deviations in parentheses).

(A) duplex-GΨC

Base pair	Shear [Å]	Stretch [Å]	Stagger [Å]	Buckle [°]	Propeller [°]	Opening [°]
U1-A18	-0.23 (0.01)	0.03 (0.00)	-0.15 (0.01)	2.3 (0.7)	-15.1 (0.2)	1.0 (0.2)
C2-G17	0.20 (0.00)	-0.01 (0.00)	0.20 (0.01)	2.6 (0.4)	-12.7 (0.7)	1.0 (0.1)
A3-U16	0.07 (0.01)	-0.01 (0.00)	0.13 (0.02)	1.0 (0.6)	-12.4 (0.6)	2.2 (0.2)
G4-C15	-0.24 (0.00)	-0.03 (0.00)	0.18 (0.01)	2.9 (0.4)	-11.2 (0.8)	1.1 (0.1)
**Ψ5-A14**	-0.17 (0.01)	-0.02 (0.00)	0.13 (0.03)	-1.0 (0.6)	-9.6 (0.6)	-0.9 (0.2)
C6-G13	0.20 (0.01)	-0.02 (0.00)	0.22 (0.01)	-2.0 (0.8)	-10.6 (0.5)	1.0 (0.1)
A7-U12	0.13 (0.01)	0.00 (0.00)	0.16 (0.01)	-0.7 (0.1)	-11.8 (0.6)	1.4 (0.1)
G8-C11	-0.27 (0.00)	-0.05 (0.00)	0.08 (0.01)	-5.7 (0.3)	-19.6 (0.2)	1.3 (0.1)
U9-A10	-0.13 (0.00)	0.03 (0.00)	0.17 (0.01)	-7.4 (0.3)	-14.8 (0.2)	0.6 (0.1)
AVG_all*	-0.05 (0.19)	-0.01 (0.02)	0.12 (0.11)	-0.9 (3.5)	-13.1 (2.9)	1.0 (0.8)
MD#	-0.02 (0.35)	0.02 (0.14)	0.04 (0.44)	1.3 (10.5)	-14.1 (8.0)	1.8 (5.1)

(B) duplex-CΨG

Base pair	Shear [Å]	Stretch [Å]	Stagger [Å]	Buckle [°]	Propeller [°]	Opening [°]
U1-A18	-0.24 (0.02)	0.04 (0.00)	0.03 (0.04)	4.4 (1.1)	-13.6 (0.9)	0.6 (0.5)
C2-G17	0.21 (0.00)	-0.01 (0.00)	0.14 (0.02)	6.8 (0.6)	-14.0 (0.2)	1.0 (0.1)
A3-U16	0.14 (0.01)	-0.01 (0.00)	0.04 (0.01)	1.8 (0.4)	-14.8 (0.3)	1.4 (0.1)
C4-G15	0.23 (0.00)	-0.02 (0.00)	0.11 (0.02)	1.5 (0.4)	-13.6 (0.5)	1.3 (0.1)
**Ψ5-A14**	-0.23 (0.01)	-0.02 (0.00)	0.12 (0.01)	-1.6 (0.3)	-11.2 (0.7)	-0.1 (0.2)
G6-C13	-0.20 (0.01)	-0.02 (0.00)	0.21 (0.00)	-1.7 (0.4)	-11.1 (0.5)	0.6 (0.1)
A7-U12	0.05 (0.01)	-0.01 (0.00)	0.14 (0.01)	-2.1 (0.3)	-13.9 (0.2)	1.9 (0.2)
G8-C11	-0.26 (0.00)	-0.03 (0.00)	0.19 (0.01)	-3.1 (0.7)	-15.4 (0.8)	1.3 (0.0)
U9-A10	-0.16 (0.01)	0.02 (0.00)	0.02 (0.02)	-1.6 (1.1)	-13.0 (0.7)	0.1 (0.3)
AVG_all*	-0.05 (0.19)	-0.01 (0.02)	0.11 (0.07)	0.5 (3.2)	-13.4 (1.5)	0.9 (0.7)
MD#	-0.01 (0.35)	0.02 (0.13)	0.0 (0.44)	0.5 (11.5)	-14.7 (7.9)	1.9 (5.2)

* Averaged over 9 base-pair and 10 models.

\# Average values were calculated using the 7 central base pairs (bp) (excluding terminal bp) and over 100-500 ns of the trajectories.

Calculations were performed with CURVES+ Version 2.6/CANAL Version 1.3.

Supplementary Table S6. Local base-pair step parameters in solution NMR structures for (A) duplex-GΨC and (B) duplex-CΨG averaged over 10 models (standard deviations in parentheses).

duplex-GΨC

Base pair step	Shift [Å]	Slide [Å]	Rise [Å]	Tilt [°]	Roll [°]	Twist [°]
U1-A18/C2-G17	0.34 (0.01)	-1.53 (0.02)	3.29 (0.00)	-1.7 (0.1)	9.2 (0.1)	32.9 (0.2)
C2-G17/A3-U16	0.00 (0.03)	-1.42 (0.02)	3.18 (0.02)	0.2 (0.2)	11.2 (0.7)	33.6 (0.3)
A3-U16/G4-C15	-0.12 (0.02)	-1.82 (0.01)	3.15 (0.01)	-1.9 (0.2)	6.2 (0.5)	29.5 (0.3)
G4-C15/**Ψ5**-A14	-0.01 (0.03)	-2.26 (0.02)	3.37 (0.01)	-0.8 (0.2)	3.4 (0.1)	30.3 (0.3)
**Ψ5**-A14/C6-G13	0.24 (0.03)	-2.02 (0.02)	3.24 (0.02)	1.6 (0.2)	5.7 (0.6)	31.7 (0.2)
C6-G13/A7-U12	-0.05 (0.01)	-1.48 (0.02)	3.15 (0.02)	1.1 (0.2)	8.5 (0.5)	32.6 (0.2)
A7-U12/G8-C11	-0.05 (0.01)	-1.48 (0.02)	3.15 (0.02)	1.1 (0.2)	8.5 0.5)	32.6 (0.2)
G8-C11/U9-A10	-0.00 (0.01)	-1.30 (0.01)	3.27 (0.01)	-0.9 (0.1)	7.3 (0.0)	34.5 (0.0)
AVG_all*	0.04 (0.15)	-1.66 (0.32)	3.24 (0.08)	-0.2 (1.2)	7.6 (2.3)	32.1 (1.6)
MD#	0.02 (0.74)	-1.60 (0.48)	3.30 (0.33)	-0.1 (4.5)	9.5 (6.8)	30.6 (3.8)

(A) duplex-CΨG

Base pair step	Shift [Å]	Slide [Å]	Rise [Å]	Tilt [°]	Roll [°]	Twist [°]
U1-A18/C2-G17	0.16 (0.04)	-1.64 (0.02)	3.23 (0.04)	-1.0 (0.4)	7.3 (0.7)	32.1 (0.5)
C2-G17/A3-U16	-0.03 (0.01)	-1.50 (0.01)	3.29 (0.01)	0.5 (0.2)	12.2 (0.3)	33.2 (0.1)
A3-U16/C4-G15	0.02 (0.02)	-1.89 (0.01)	3.29 (0.01)	-0.6 (0.1)	4.1 (0.1)	31.0 (0.2)
C4-G15/**Ψ5**-A14	0.09 (0.01)	-1.89 (0.02)	3.28 (0.01)	0.9 (0.2)	7.1 (0.9)	29.3 (0.4)
**Ψ5**-A14/G6-C13	0.07 (0.03)	-1.60 (0.02)	3.20 (0.01)	-1.2 (0.2)	8.3 (0.5)	32.5 (0.3)
G6-C13/A7-U12	-0.11 (0.01)	-1.82 (0.03)	3.24 (0.00)	0.0 (0.1)	6.6 (0.3)	31.4 (0.3)
A7-U12/G8-C11	-0.20 (0.02)	-1.75 (0.02)	3.20 (0.02)	-1.3 (0.1)	6.7 (0.4)	31.0 (0.2)
G8-C11/U9-A10	0.07 (0.01)	-1.84 (0.02)	3.27 (0.01)	1.9 (0.1)	3.5 (0.2)	32.0 (0.3)
AVG_all*	0.01 (0.11)	-1.74 (0.14)	3.25 (0.04)	-0.1 (1.1)	7.0 (2.5)	31.6 (1.1)
MD#	0.02 (0.71)	-1.58 (0.47)	3.33 (0.34)	-0.1 (4.4)	9.7 (7.0)	30.7 (3.9)

* Averaged over 9 base-pair and 10 models.

# Average values were calculated using the 7 central base pairs (bp) (excluding terminal bp) and over 100-500 ns of the trajectories.

Calculations were performed with CURVES+ Version 2.6/CANAL Version 1.3.

Supplementary Table S7. Average mass-weighted root-mean-square deviations (RMSDs) and radius of gyrations (R_Gs) for the studied 9-bp duplexes*.

	duplex-GΨC (duplex-GUC)	duplex-CΨG (duplex-CUG)	duplex-AΨU (duplex-AUU)	duplex-UΨA (duplex-UUA)
RMSD (Å)	1.39 ± 0.26<br>*1.20 ± 0.33*<br>(1.41 ± 0.27)	1.39 ± 0.26<br>*1.21 ± 0.38*<br>(1.43 ± 0.27)	1.40 ± 0.26<br><br>(1.42 ± 0.29)	1.39 ± 0.27<br><br>(1.47 ± 0.33)
R_G (Å)	10.94 ± 0.28<br>(10.92 ± 0.28)	10.82 ± 0.31<br>(10.81 ± 0.29)	10.79 ± 0.27<br>(10.87 ± 0.28)	10.75 ± 0.27<br>(10.79 ± 0.26)

*Averages and standard deviation were calculated over 100-500 ns of the trajectories for heavy atoms in reference to the initial A-form geometry and additionally for duplex-GΨC and duplex-CΨG in reference to the NMR model (in italic). Average values for unmodified duplexes are in parentheses.

Supplementary Table S8. The representative average structural parameters in studied RNA duplexes calculated from MD simulations (standard deviations in parentheses).

	duplex-GΨC		duplex-CΨG		duplex-AΨU		duplex-UΨA		A-RNA*	RNA#
	mod	ref	mod	ref	mod	ref	mod	ref		
BP-Axis										
Inclination [°]	16.6 *(5.7)*	16.7 *(5.8)*	17.1 *(5.8)*	17.2 *(5.8)*	18.1 *(6.0)*	19.5 *(6.1)*	18.6 *(6.0)*	19.6 *(6.3)*	15.9	
Intra-BP										
Buckle [°]	1.3 *(10.5)*	1.7 *(10.7)*	0.5 *(11.5)*	1.0 *(11.6)*	0.6 *(11.6)*	1.0 *(11.7)*	1.3 *(11.5)*	1.5 *(10.7)*	0.0	
Propeller [°]	-14.1 *(8.0)*	-14.3 *(8.1)*	-14.7 *(7.9)*	-15.1 *(7.9)*	-15.4 *(8.5)*	-16.0 *(8.4)*	-15.9 *(8.2)*	-16.2 *(8.3)*	13.7	
Opening [°]	1.8 *(5.1)*	2.2 *(5.1)*	1.9 *(5.2)*	2.4 *(5.1)*	2.3 *(5.6)*	2.8 *(5.6)*	2.3 *(5.8)*	2.8 *(5.6)*	-3.6	
Inter-BP										
Shift (Dx) [Å]	0.02 *(0.74)*	0.02 *(0.70)*	0.02 *(0.71)*	0.03 *(0.70)*	0.03 *(0.71)*	0.02 *(0.70)*	0.02 *(0.69)*	0.03 *(0.67)*	0.0	0.0 *(0.12)*
Slide (Dy) [Å]	-1.60 *(0.48)*	-1.59 *(0.48)*	-1.58 *(0.47)*	-1.56 *(0.47)*	-1.51 *(0.48)*	-1.44 *(0.47)*	-1.49 *(0.48)*	-1.47 *(0.48)*	-1.69	-1.44 *(0.15)*
Roll [°]	9.5 *(6.8)*	9.5 *(6.9)*	9.7 *(7.0)*	9.8 *(7.0)*	10.3 *(7.1)*	11.1 *(7.3)*	10.7 *(7.5)*	11.2 *(7.7)*	9.0	7.91 *(2.11)*
Twist [°]	30.6 *(3.8)*	30.5 *(4.5)*	30.7 *(3.9)*	30.6 *(3.7)*	30.3 *(3.8)*	30.3 *(3.8)*	30.5 *(4.3)*	30.3 *(4.3)*	31.4	32.29 *(1.93)*
h-rise [Å]	2.69 *(0.41)*	2.69 *(0.43)*	2.70 (0.42)	2.69 *(0.41)*	2.66 *(0.43)*	2.61 *(0.44)*	2.64 *(0.45)*	2.60 *(0.46)*	2.81	

mod – modified duplex, ref – unmodified reference duplex.

Average values were calculated using the 7 central base pairs (bp) (excluding terminal bp) and over 100-500 ns of the trajectories. Standard deviations *(SD)* reflect the range of thermal fluctuations in the MD simulations.

* A-RNA was generated by NAB in the Amber Tools15 based on the fibre-diffraction data [1].

\# data from X-ray structures database, analyzed with Curves+ [2].

A-RNA generated by the NAB has positive propeller and negative opening whereas structures from MD display negative propeller and positive opening. Negative propeller and positive opening is also reported for experimental structures [3,4], analyzed with NUPARM and 3DNA program, respectively.

Supplementary Table S9. Interaction energies in the eight base-pair steps containing the Ψ-A base pair [kcal/mol]. For the comparison interaction energies were calculated for similar systems without Ψ modification. Interaction energies were calculated both with molecular mechanics (MM) force field and at the quantum-chemical (QM) level (B97D/Def2TZVPP level of theory). MM energies (decomposed into van der Waals and electrostatic terms) were calculated using 40000 frames from 100-500 ns of the molecular dynamics trajectories. QM calculations are based on the geometries of the average structure of the most populated clusters obtained from the cluster analysis of the appropriate trajectory. QM energies are shown in blue.

duplex-GΨC			Modified duplex			Reference duplex		
			Coulomb (kcal/mol)	VDW (kcal/mol)	Total (kcal/mol)	Coulomb (kcal/mol)	VDW (kcal/mol)	Total (kcal/mol)
Stack 1<br>4 5<br>5' - G Ψ - 3'<br>3' - C A - 5'<br>15 14	Intra-strand	4G/5Ψ	0.95 (1.25)	-5.89 (0.61)	-4.94 (1.39)<br>**-4.40**	2.08 (1.32)	-6.07 (0.62)	-3.99 (1.46)<br>**-3.73**
		14A/15C	1.30 (0.89)	-5.36 (0.69)	-4.06 (1.13)<br>**-3.76**	1.30 (0.87)	-5.33 (0.67)	-4.03 (1.10)<br>**-3.68**
	Inter-strand	4G/14A	-1.71 (1.03)	-1.01 (0.41)	-2.72 (1.11)<br>**-3.59**	-1.68 (1.13)	-1.07 (0.42)	-2.75 (1.21)<br>**-3.45**
		5Ψ/15C	2.81 (1.21)	-1.67 (0.61)	1.14 (1.36)<br>**-0.73**	2.79 (1.26)	-1.59 (0.60)	1.20 (1.40)<br>**-1.14**
	**Step stacking (sum)**	**4G-15C/5Ψ-14A**	**3.35 (2.21)**	**-13.93 (1.18)**	**-10.58 (2.51)**<br>**-12.48**	**4.49 (2.32)**	**-14.06 (1.17)**	**-9.57 (2.6)**<br>**-12.00**
	Inter-strand (base pair)	4G-15C	-26.37 (2.58)	0.44 (1.72)	-25.93 (3.10)<br>**-23.18**	-26.49 (2.58)	0.49 (1.72)	-26.0 (3.10)<br>**-23.31**
		5Ψ-14A	-8.47 (2.07)	-0.52 (1.31)	-8.99 (2.45)<br>**-12.33**	-8.66 (2.11)	-0.58 (1.30)	-9.24 (2.48)<br>**-12.66**
	**Step base pair (sum)**	**4G-15C/5Ψ-14A**	**-34.84 (3.31)**	**-0.08 (2.16)**	**-34.92 (3.95)**<br>**-35.51**	**-35.15 (3.33)**	**-0.09 (2.16)**	**-35.24 (3.97)**<br>**-35.97**
	**Step all (sum)**	**4G-15C/5Ψ-14A**	**-31.49 (3.98)**	**-14.01 (2.46)**	**-45.50 (4.68)**<br>**-47.99**	**-30.66 (4.06)**	**-14.15 (2.46)**	**-44.81 (4.75)**<br>**-47.97**
	Step (combined)	4G-15C-5Ψ-14A			**-47.36**			**-47.17**
**QM stacking**					**-11.85**			**-11.20**
Stack 2<br>5 6<br>5' - Ψ C - 3'<br>3' - A G - 5'<br>14 13	Intra-strand	5Ψ/6C	0.18 (1.01)	-3.91 (0.56)	-3.73 (1.15)<br>**-4.24**	-0.22 (1.05)	-3.90 (0.58)	-4.12 (1.20)<br>**-4.61**
		13G/14A	-1.73 (1.49)	-6.82 (0.70)	-8.55 (1.65)<br>**-7.55**	-1.49 (1.47)	-6.89 (0.69)	-8.38 (1.62)<br>**-7.58**
	Inter-strand	5Ψ/13G	1.65 (0.55)	-0.40 (0.14)	1.25 (0.57)<br>**0.79**	2.19 (0.64)	-0.41 (0.15)	1.78 (0.66)<br>**1.16**
		6C/14A	4.36 (0.77)	-3.17 (0.58)	1.19 (0.96)<br>**-0.99**	4.38 (0.79)	-3.11 (0.58)	1.27 (0.98)<br>**-0.94**
	**Step stacking (sum)**	**5Ψ-14A/6C-13G**	**4.46 (2.03)**	**-14.3 (1.08)**	**-9.84 (2.3)**<br>**-11.99**	**4.86 (2.07)**	**-14.31 (1.08)**	**-9.45 (2.33)**<br>**-12.66**
	Inter-strand (base pair)	5Ψ-14A	-8.47 (2.07)	-0.52 (1.31)	-8.99 (2.45)<br>**-12.33**	-8.66 (2.11)	-0.58 (1.30)	-9.24 (2.48)<br>**-12.66**
		6C-13G	-26.39 (2.48)	0.29 (1.68)	-26.10 (3.00)<br>**-23.93**	-26.39 (2.51)	0.31 (1.69)	-26.08 (3.03)<br>**-24.02**
	**Step base pair (sum)**	**5Ψ-14A/6C-13G**	**-34.86 (3.23)**	**-0.23 (2.13)**	**-35.09 (3.87)**<br>**-36.26**	**-35.05 (3.28)**	**-0.27 (2.13)**	**-35.32 (3.91)**<br>**-36.68**
	**Step all (sum)**	**5Ψ-14A/6C-13G**	**-30.4 (3.81)**	**-14.53 (2.39)**	**-44.93 (4.50)**<br>**-48.25**	**-30.19 (3.88)**	**-14.58 (2.39)**	**-44.77 (4.56)**<br>**48.65**
	Step (combined)	5Ψ-14A-6C-13G			**-47.76**			**-48.22**
**QM stacking**					**-11.50**			**-11.54**

duplex-CΨG			Modified duplex			Reference duplex		
			Coulomb (kcal/mol)	VDW (kcal/mol)	Total (kcal/mol)	Coulomb (kcal/mol)	VDW (kcal/mol)	Total (kcal/mol)
Stack 1<br><br>4 5<br>5' - C Ψ - 3'<br>3' - G A - 5'<br>15 14	Intra-strand	4C/5Ψ	0.82 (0.87)	-3.65 (0.59)	-2.83 (1.05)<br>**-4.25**	-0.32 (0.97)	-3.84 (0.60)	-4.16 (1.14)<br>**-5.61**
		14A/15G	1.04 (1.01)	-6.09 (0.69)	-5.05 (1.22)<br>**-6.31**	1.08 (1.06)	-6.01 (0.73)	-4.93 (1.29)<br>**-6.07**
	Inter-strand	4C/14A	1.37 (0.27)	-0.41 (0.16)	0.96 (0.31)<br>**0.22**	1.41 (0.28)	-0.44 (0.17)	0.97 (0.33)<br>**0.25**
		5Ψ/15G	2.83 (1.00)	-3.81 (0.64)	-0.98 (1.19)<br>**-1.89**	3.55 (1.12)	-3.77 (0.65)	-0.22 (1.29)<br>**-0.88**
	**Step stacking (sum)**	**4C-15G/5Ψ-14A**	**6.06 (1.69)**	**-13.96 (1.12)**	**-7.9 (2.03)**<br>**-12.23**	**5.72 (1.84)**	**-14.06 (1.16)**	**-8.34 (2.18)**<br>**-12.31**
	Inter-strand (base pair)	4C-15G	-26.26 (2.57)	0.32 (1.69)	-25.94 (3.08)<br>**-23.57**	-26.44 (2.56)	0.45 (1.72)	-25.99 (3.08)<br>**-23.42**
		5Ψ-14A	-9.09 (1.79)	-0.37 (1.34)	-9.46 (2.24)<br>**-12.38**	-9.25 (1.95)	-0.40 (1.34)	-9.65 (2.37)<br>**-12.77**
	**Step base pair (sum)**	**4C-15G/5Ψ-14A**	**-35.35 (3.13)**	**-0.05 (2.16)**	**-35.4 (3.80)**<br>**-35.95**	**-35.69 (3.22)**	**0.05 (2.18)**	**-35.64 (3.89)**<br>**-36.19**
	**Step all (sum)**	**4C-15G/5Ψ-14A**	**-29.29 (3.56)**	**-14.01 (2.43)**	**-43.30 (4.31)**<br>**-48.18**	**-29.97 (3.71)**	**-14.01 (2.47)**	**-43.98 (4.46)**<br>**-48.50**
	Step (combined)	4C-15G-5Ψ-14A			**-46.64**			**-46.97**
**QM stacking**					**-10.69**			**-10.78**
Stack 2<br><br>5 6<br>5' - Ψ G - 3'<br>3' - A C - 5'<br>14 13	Intra-strand	5Ψ/6G	2.24 (1.11)	-4.21 (0.62)	-1.97 (1.27)<br>**-4.04**	3.65 (1.28)	-4.09 (0.69)	-0.44 (1.45)<br>**-2.39**
		13C/14A	2.35 (0.62)	-4.08 (0.61)	-1.73 (0.87)<br>**-3.31**	2.32 (0.62)	-4.13 (0.61)	-1.81 (0.87)<br>**-3.60**
	Inter-strand	5Ψ/13C	0.11 (0.31)	-0.20 (0.06)	-0.09 (0.32)<br>**-0.92**	-0.07 (0.35)	-0.20 (0.07)	-0.27 (0.36)<br>**-1.16**
		6G/14A	-0.78 (1.28)	-5.67 (0.73)	-6.45 (1.47)<br>**-6.05**	-0.41 (1.41)	-5.71 (0.74)	-6.12 (1.59)<br>**-5.56**
	**Step stacking (sum)**	**5Ψ-14A/6G-13C**	**3.92 (1.83)**	**-14.16 (1.14)**	**-10.24 (2.16)**<br>**-14.32**	**5.49 (2.03)**	**-14.13 (1.18)**	**-8.64 (2.35)**<br>**-12.71**
	Inter-strand (base pair)	5Ψ-14A	-9.09 (1.79)	-0.37 (1.34)	-9.46 (2.24)<br>**-12.38**	-9.25 (1.95)	-0.40 (1.34)	-9.65 (2.37)<br>**-12.77**
		6G-13C	-26.24 (2.50)	0.21 (1.66)	-26.24 (2.50)<br>**-24.06**	-26.33 (2.50)	0.28 (1.68)	-26.05 (3.01)<br>**-24.06**
	**Step base pair (sum)**	**5Ψ-14A/6G-13C**	**-35.33 (3.07)**	**-0.16 (2.13)**	**-35.49 (3.74)**<br>**-36.44**	**-35.58 (3.17)**	**-0.12 (2.15)**	**-35.7 (3.83)**<br>**-36.83**
	**Step all (sum)**	**5Ψ-14A/6G-13C**	**-29.29 (3.56)**	**-14.01 (2.43)**	**-45.73 (4.31)**<br>**-50.76**	**-30.09 (3.76)**	**-14.25 (2.45)**	**-44.34 (4.49)**<br>**-49.54**
	Step (combined)	**5Ψ-14A-6G-13C**			**-49.56**			**-48.36**
**QM stacking**					**-13.12**			**-11.53**

duplex-AΨU			Modified duplex			Reference duplex		
			Coulomb (kcal/mol)	VDW (kcal/mol)	Total (kcal/mol)	Coulomb (kcal/mol)	VDW (kcal/mol)	Total (kcal/mol)
Stack 1<br><br>4 5<br>5' - A Ψ - 3'<br>3' - U A - 5'<br>15 14	Intra-strand	4A/5Ψ	0.29 (0.88)	-5.28 (0.66)	-4.99 (1.10)<br>**-5.14**	0.40 (0.88)	-5.37 (0.68)	-4.97 (1.11)<br>**-5.36**
		14A/15U	0.25 (0.87)	-5.43 (0.66)	-5.18 (1.09)<br>**-5.66**	0.37 (0.89)	-5.42 (0.68)	-5.05 (1.12)<br>**-5.61**
	Inter-strand (cross-pair)	4A/14A	0.95 (0.34)	-1.03 (0.33)	-0.08 (0.47)<br>**-0.96**	1.00 (0.36)	-1.11 (0.35)	-0.11 (0.50)<br>**-1.07**
		5Ψ/15U	3.43 (1.27)	-1.57 (0.50)	1.86 (1.36)<br>**0.62**	3.46 (1.28)	-1.45 (0.50)	2.01 (1.37)<br>**0.60**
	**Step stacking (sum)**	**4A-15U/5U-14A**	**4.92 (1.81)**	**-13.31 (1.11)**	**-8.39 (2.12)**<br>**-11.14**	**5.23 (1.83)**	**-13.35 (1.14)**	**-8.12 (2.16)**<br>**-11.44**
	Inter-strand (base pair)	4A-15U	-8.80 (2.02)	-0.58 (1.30)	-9.38 (2.40)<br>**-12.47**	-8.75 (2.12)	-0.56 (1.30)	-9.31 (2.49)<br>**-12.39**
		5Ψ-14A	-8.64 (1.92)	-0.51 (1.31)	-9.15 (2.32)<br>**-12.02**	-8.90 (1.99)	-0.51 (1.32)	-9.41 (2.39)<br>**-12.19**
	**Step base pair (sum)**	**4A-15U/5Ψ-14A**	**-17.44 (2.79)**	**-1.09 (1.85)**	**-18.53 (3.35)**<br>**-24.49**	**-17.65 (2.91)**	**-1.07 (1.85)**	**-18.72 (3.45)**<br>**-24.58**
	**Step all (sum)**	**4A-15U/5Ψ-14A**	**-12.52 (3.33)**	**-14.4 (2.16)**	**-26.92 (3.97)**<br>**-35.63**	**-12.42 (3.44)**	**-14.42 (2.17)**	**-26.84 (4.07)**<br>**-36.02**
	Step (combined)	4A-15U-5Ψ-14A			**-34.39**			**-34.71**
**QM stacking**					**-9.90**			**-10.13**
Stack 2<br><br>5 6<br>5' - Ψ U - 3'<br>3' - A A - 5'<br>14 13	Intra-strand	5Ψ/6U	2.87 (0.88)	-3.97 (0.55)	-1.10 (1.04)<br>**-2.28**	3.73 (0.95)	-3.94 (0.60)	-0.21 (1.12)<br>**-1.29**
		13A/14A	1.65 (0.70)	-5.97 (0.71)	-4.32 (1.00)<br>**-4.45**	1.74 (0.72)	-5.97 (0.73)	-4.23 (1.03)<br>**-4.20**
	Inter-strand	5Ψ/13A	0.21 (0.36)	-0.42 (0.16)	-0.21 (0.39)<br>**-1.31**	0.16 (0.42)	-0.44 (0.17)	-0.28 (0.45)<br>**-1.64**
		6U/14A	0.95 (0.85)	-3.00 (0.63)	-2.05 (1.06)<br>**-2.65**	1.07 (0.90)	-2.94 (0.62)	-1.87 (1.09)<br>**-2.51**
	**Step stacking (sum)**	**5Ψ-14A/6U-13A**	**5.68 (1.45)**	**-13.36 (1.11)**	**-7.68 (1.83)**<br>**-10.69**	**6.70 (1.55)**	**-13.29 (1.14)**	**-6.59 (1.92)**<br>**-9.64**
	Inter-strand (base pair)	5Ψ-14A	-8.64 (1.92)	-0.51 (1.31)	-9.15 (2.32)<br>**-12.02**	-8.90 (1.99)	-0.51 (1.32)	-9.41 (2.39)<br>**-12.19**
		6U-13A	-9.07 (1.87)	-0.64 (1.27)	-9.71 (2.26)<br>**-12.77**	-9.06 (1.87)	-0.61 (1.28)	-9.67 (2.27)<br>**-12.65**
	**Step base pair (sum)**	**5Ψ-14A/6U-13A**	**-17.71 (2.68)**	**-1.15 (1.82)**	**-18.86 (3.24)**<br>**-24.79**	**-17.96 (2.73)**	**-1.12 (1.84)**	**-19.08 (3.29)**<br>**-24.84**
	**Step all (sum)**	**5Ψ-14A/6U-13A**	**-12.03 (3.05)**	**-14.51 (2.13)**	**-26.54 (3.72)**<br>**-35.48**	**-11.26 (3.14)**	**-14.41 (2.16)**	**-25.67 (3.81)**<br>**-34.48**
	Step (combined)	5Ψ-14A-6U-13A			**-35.06**			**-34.03**
**QM stacking**					**-10.27**			**-9.19**

duplex-UΨA			Modified duplex			Reference duplex		
			Coulomb (kcal/mol)	VDW (kcal/mol)	Total (kcal/mol)	Coulomb (kcal/mol)	VDW (kcal/mol)	Total (kcal/mol)
Stack 1<br><br>4 5<br>5' - U Ψ - 3'<br>3' - A A - 5'<br>15 14	Intra-strand	4U/5Ψ	3.79 (0.95)	-3.80 (0.59)	-0.01 (1.12)<br>**-1.12**	3.80 (0.96)	-3.94 (0.60)	-0.14 (1.13)<br>**-1.29**
		14A/15A	1.73 (0.71)	-6.01 (0.73)	-4.28 (1.02)<br>**-4.54**	1.74 (0.71)	-5.97 (0.74)	-4.23 (1.03)<br>**-4.24**
	Inter-strand	4U/14A	0.15 (0.41)	-0.43 (0.17)	-0.28 (0.44)<br>**-1.55**	0.12 (0.42)	-0.45 (0.17)	-0.33 (0.45)<br>**-1.59**
		5Ψ/15A	1.43 (0.82)	-2.99 (0.61)	-1.56 (1.02)<br>**-2.33**	1.13 (0.89)	-2.93 (0.62)	-1.80 (1.08)<br>**-2.50**
	**Step stacking (sum)**	**4U-15A/5Ψ-14A**	**7.10 (1.50)**	**-13.23 (1.13)**	**-6.13 (1.88)**<br>**-9.54**	**6.79 (1.55)**	**-13.29 (1.15)**	**-6.5 (1.93)**<br>**-9.62**
	Inter-strand (base pair)	4U-15A	-8.75 (2.00)	-0.61 (1.28)	-9.36 (2.37)<br>**-12.23**	-8.82 (1.98)	-0.56 (1.30)	-9.38 (2.37)<br>**-12.19**
		5Ψ-14A	-8.77 (1.84)	-0.55 (1.29)	-9.32 (2.25)<br>**-12.17**	-9.03 (1.90)	-0.60 (1.28)	-9.63 (2.29)<br>**-12.63**
	**Step base pair (sum)**	**4U-15A/5Ψ-14A**	**-17.52 (2.72)**	**-1.16 (1.82)**	**-18.68 (3.27)**<br>**-24.40**	**-17.85 (2.74)**	**-1.16 (1.82)**	**-19.01 (3.29)**<br>**-24.82**
	**Step all (sum)**	**4U-15A/5Ψ-14A**	**-10.42 (3.11)**	**-14.39 (2.14)**	**-24.81 (3.77)**<br>**-33.94**	**-11.06 (3.15)**	**-14.45 (2.15)**	**-25.51 (3.81)**<br>**-34.44**
	Step (combined)	4U-15A-5Ψ-14A			**-33.61**			**-33.99**
**QM stacking**					**-9.21**			**-9.17**
Stack 2<br><br>5 6<br>5' - Ψ A - 3'<br>3' - A U - 5'<br>14 13	Intra-strand	5Ψ/6A	-1.28 (1.34)	-4.15 (0.61)	-5.43 (2.17)<br>**-5.72**	-0.91 (1.43)	-4.07 (0.62)	-4.98 (1.56)<br>**-5.03**
		13U/14A	-0.73 (1.31)	-4.10 (0.62)	-4.83 (2.10)<br>**-5.01**	-0.79 (1.36)	-4.10 (0.63)	-4.89 (1.50)<br>**-4.96**
	Inter-strand	5Ψ/13U	1.90 (0.31)	-0.19 (0.06)	1.71 (0.32)<br>**0.71**	2.31 (0.35)	-0.19 (0.06)	2.12 (0.36)<br>**0.90**
		6A/14A	2.01 (0.50)	-4.75 (0.74)	-2.74 (0.89)<br>**-3.60**	2.05 (0.50)	-4.72 (0.74)	-2.67 (0.89)<br>**-3.57**
	**Step stacking (sum)**	**5Ψ-14A/6A-13U**	**1.90 (1.94)**	**-13.19 (1.14)**	**-11.29 (3.16)**<br>**-13.62**	**2.66 (2.07)**	**-13.08 (1.15)**	**-10.42 (2.37)**<br>**-12.66**
	Inter-strand (base pair)	5Ψ-14A	-8.77 (1.84)	-0.55 (1.29)	-9.32 (2.25)<br>**-12.17**	-9.03 (1.90)	-0.60 (1.28)	-9.63 (2.29)<br>**-12.63**
		6A-13U	-9.06 (1.90)	-0.62 (1.28)	-9.68 (2.29)<br>**-12.65**	-9.09 (1.87)	-0.61 (1.28)	-9.70 (2.27)<br>**-12.69**
	**Step base pair (sum)**	**5Ψ-14A/6A-13U**	**-17.83 (2.64)**	**-1.17 (1.82)**	**-19.00 (3.21)**<br>**-24.82**	**-18.12 (2.67)**	**-1.21 (1.81)**	**-19.33 (3.23)**<br>**-25.32**
	**Step all (sum)**	**5Ψ-14A/6A-13U**	**-15.93 (3.28)**	**-14.36 (2.15)**	**-30.29 (3.92)**<br>**-38.44**	**-15.46 (3.38)**	**-14.29 (2.14)**	**-29.75 (3.68)**<br>**-37.98**
	Step (combined)	5Ψ-14A-6A-13U	3.79 (0.95)	-3.80 (0.59)	-0.01<br>**-38.44**			**-38.00**
**QM stacking**					**-13.62**			**-12.68**

Supplementary Table S10. MM-PBSA binding free energies [kcal/mol], σ_M - standard error of the mean.

Energy component	Duplex-GΨC	σ_M	Duplex-GUC	σ_M	ΔΔE_(Ψ-U)
E_VDW	-48.00	*0.05*	-47.93	*0.05*	0.07
E_EL	1082.18	*0.37*	1082.74	*0.37*	-0.56
E_PB	-1102.81	*0.35*	-1104.06	*0.36*	1.25
E_NP	-6.90	*0.002*	-6.90	*0.002*	0.00
E(EL+PB)	-20.63		-21.32		0.69
ΔG_{bind}	-75.53	*0.05*	-76.14	*0.05*	0.61
TΔS	-29.78		-29.28		-0.50
$\Delta\Delta G_{bind}$					1.11
$\Delta\Delta G^{EXP,\ 5}$					-0.71 (±0.55)

	Duplex-CΨG	σ_M	Duplex-CUG	σ_M	ΔΔE_(Ψ-U)
E_VDW	-48.19	*0.05*	-48.30	*0.05*	0.11
E_EL	1075.53	*0.38*	1078.43	*0.43*	-2.90
E_PB	-1097.56	*0.36*	-1099.28	*0.40*	1.72
E_NP	-4.82	*0.002*	-4.82	*0.002*	0.00
E(EL+PB)	-22.02		-20.85		-1.17
ΔG_{bind}	-75.04	*0.05*	-73.97	*0.05*	-1.07
TΔS	-29.48		-29.58		0.10
$\Delta\Delta G_{bind}$					-1.17
$\Delta\Delta G^{EXP,\ 5}$					-2.43 (±0.49)

	Duplex-AΨU	σ_M	Duplex-AUU	σ_M	ΔΔE_(Ψ-U)
E_VDW	-47.46	*0.05*	-47.25	*0.05*	-0.21
E_EL	1114.98	*0.39*	1123.07	*0.40*	-8.09
E_PB	-1128.63	*0.37*	-1135.44	*0.38*	6.81
E_NP	-4.70	*0.002*	-4.72	*0.002*	0.02
E(EL+PB)	-13.65		-12.37		-1.28
ΔG_{bind}	-65.80	*0.05*	-64.33	*0.05*	-1.47
TΔS	-28.46		-28.50		0.04
$\Delta\Delta G_{bind}$					-1.51
$\Delta\Delta G^{EXP, 5}$					-0.27 (±0.27)

	Duplex-UΨA	σ_M	Duplex-UUA	σ_M	ΔΔE_(Ψ-U)
E_VDW	-47.36	*0.05*	-47.62	*0.05*	0.26
E_EL	1128.08	*0.39*	1129.96	*0.41*	-1.88
E_PB	-1140.09	*0.37*	-1141.43	*0.39*	1.32
E_NP	-4.70	*0.002*	-4.72	*0.003*	0.02
E(EL+PB)	-12.01		-11.47		-0.54
ΔG_{bind}	-64.06	*0.05*	-63.80	*0.05*	-0.26
TΔS	-27.72		-28.05		0.32
$\Delta\Delta G_{bind}$					-0.58
$\Delta\Delta G^{EXP, 5}$					-0.55 (±0.19)

Duplex formation energy (kcal/mol) was calculated by subtracting the energy of the two single strands from that of the duplex, using single-trajectory approach. E_VDW denotes van der Waals energy, E_EL - electrostatic energy, E_PB - electrostatic solvation free energy with the Poisson Boltzmann (PB) model, E_NP - contribution of the nonpolar surface area to the solvation free energy, TΔS configurational entropy of the solute from quasi-harmonic analysis. Calculations of ΔG_{bind} do not include contributions due to changes in the conformational entropy of the RNA. Experimental data from Kierzek at al. [5], standard deviation in parenthesis.

SUPPLEMENTARY FIGURES

Supplementary Figure S1. Mass-weighted root-mean-square deviation (RMSD) time evolutions for the duplex-GΨC and duplex-CΨG in reference to the NMR model (model 1 of ten lowest-energy NMR ensemble).

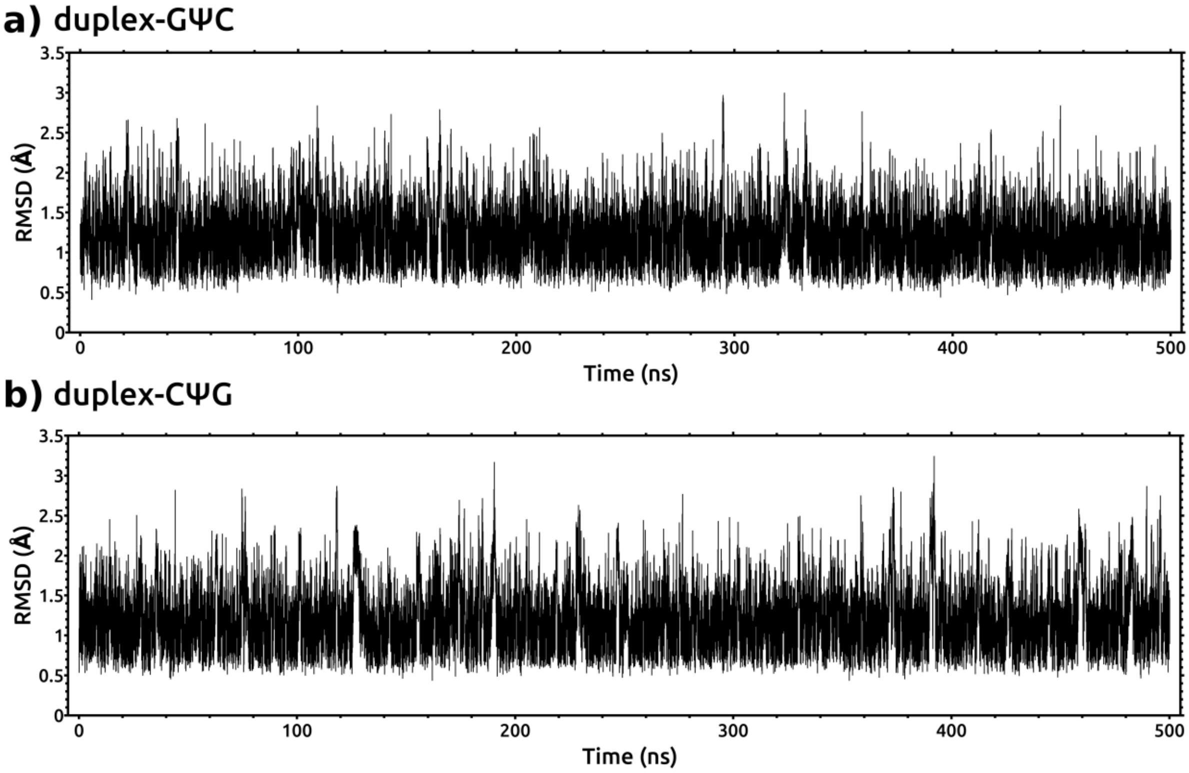

Supplementary Figure S2. Root-mean-square fluctuation (RMSF) of the backbone atoms (P, OP1, OP2, O5', C5' and O3') calculated per residue in the studied RNA duplexes, red – Ψ-modified duplex, black – reference, unmodified duplex.

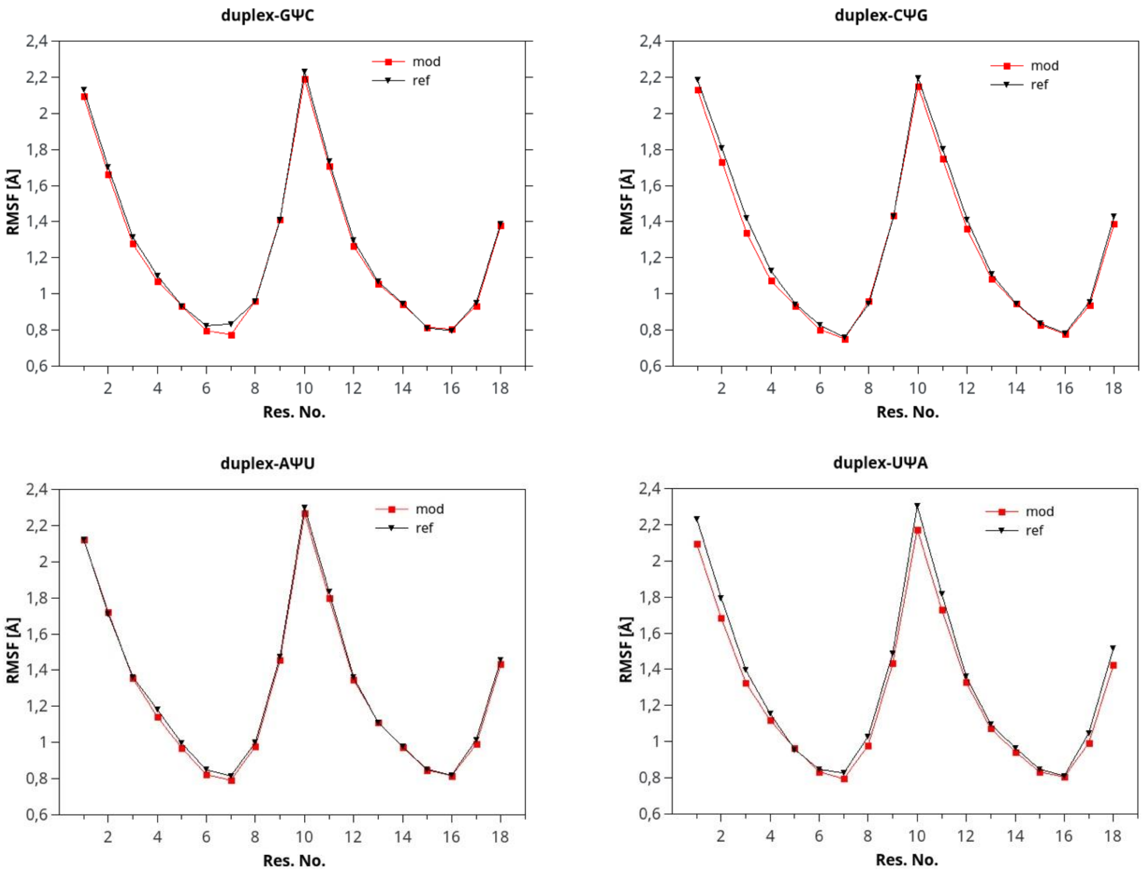

Supplementary Figure S3. S^2 order parameter of the C1'-H1' vector of the sugar moiety calculated in the studied RNA duplexes, red – Ψ-modified duplex, black – reference, unmodified duplex.

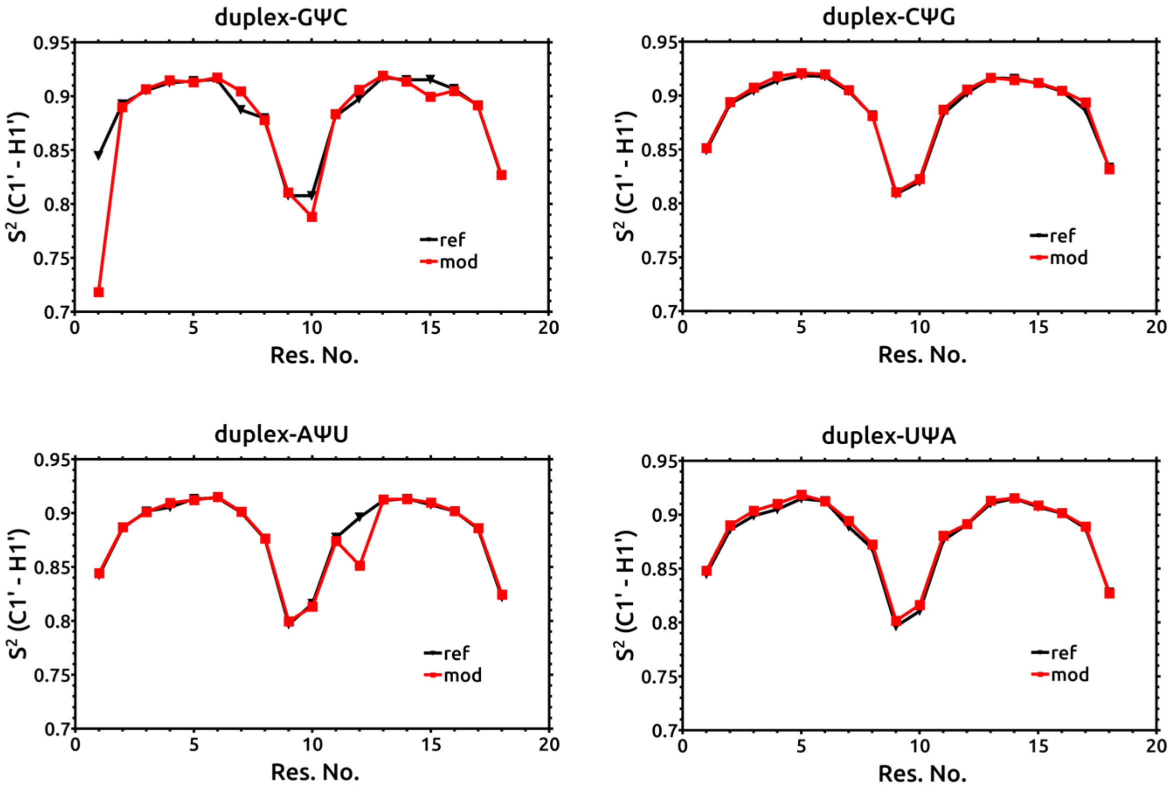

Supplementary Figure S4. Local base-pair parameters in the Ψ-modified and reference, unmodified duplexes: opening; green – Ψ-modified NMR; red – Ψ-modified from MD simulation; black – reference, unmodified from MD simulation. Vertical lines represent standard deviations.

Opening (σ)

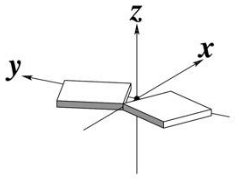

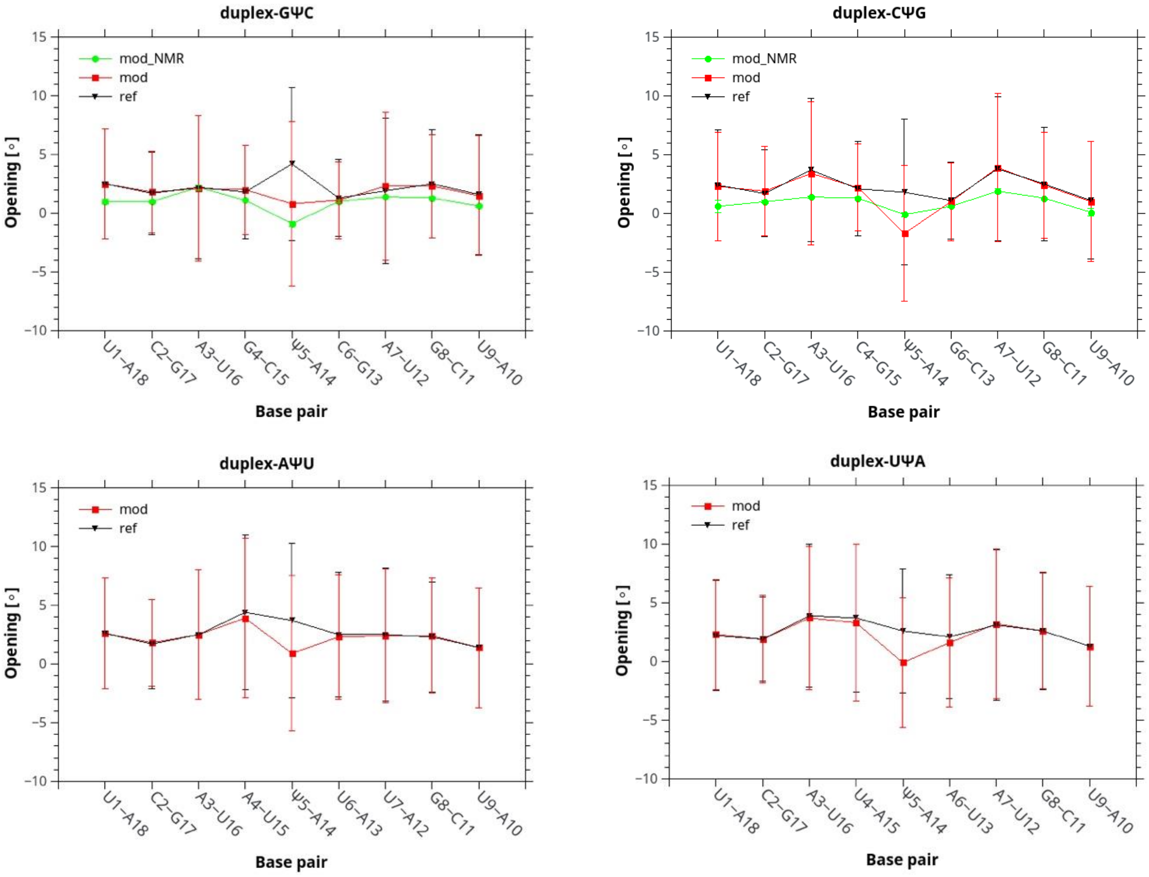

Supplementary Figure S5. Local base-pair step and helical parameters in the Ψ-modified and reference, unmodified duplexes: (A) shift, (B) roll, (C) inclination; green – Ψ-modified NMR; red – Ψ-modified from MD simulation; black – reference, unmodified from MD simulation. Vertical lines represent standard deviations.

(A) Shift (Dx)

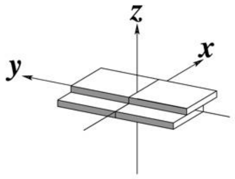

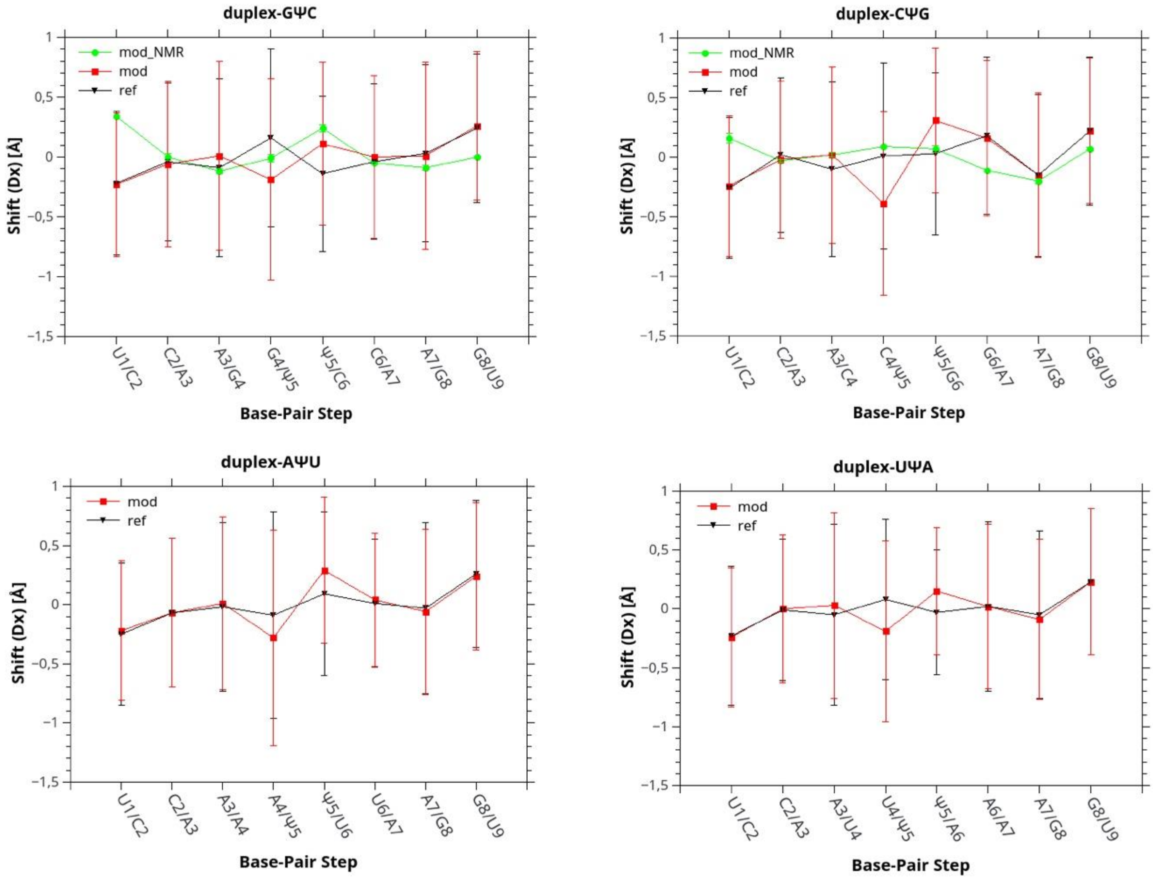

(B) Roll (ρ)

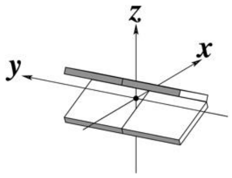
z
x
y

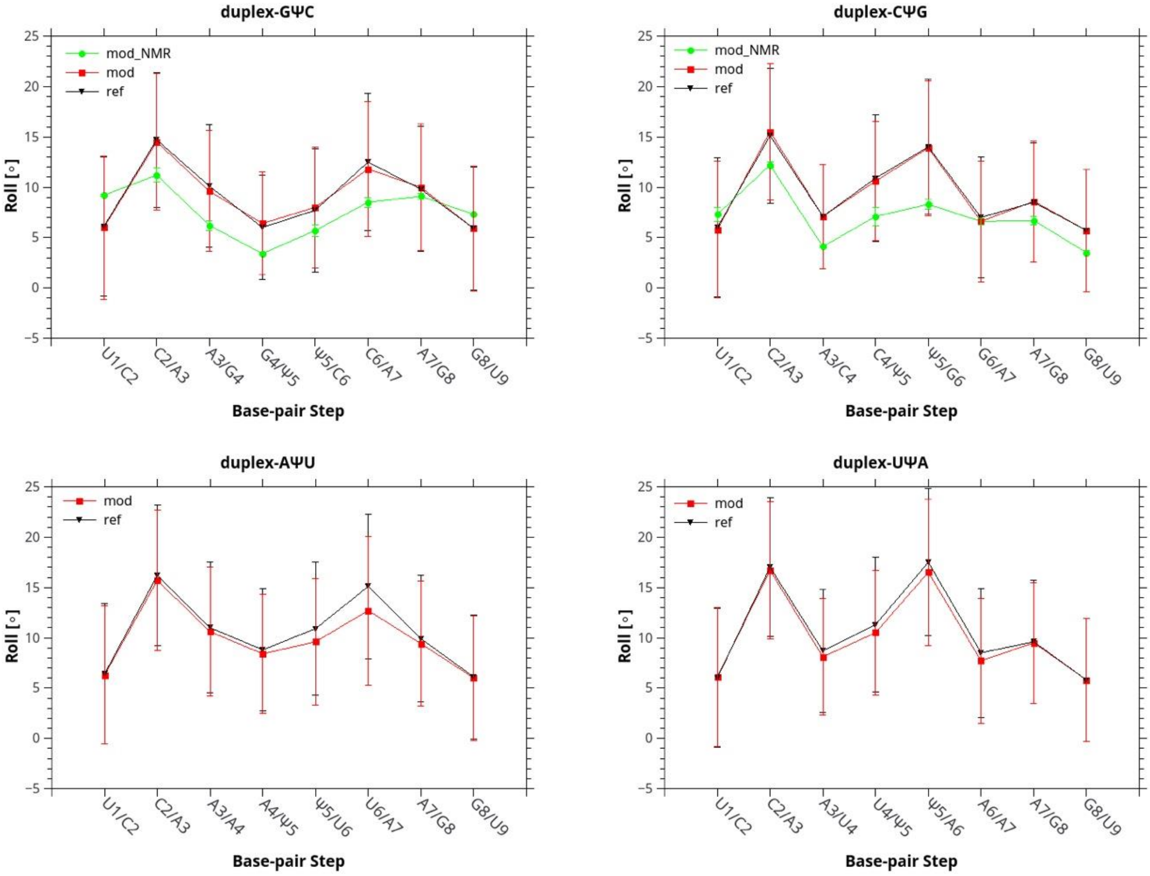
duplex-GΨC
mod_NMR
mod
ref
Roll [°]
Base-pair Step
U1/C2
C2/A3
A3/G4
G4/Ψ5
Ψ5/C6
C6/A7
A7/G8
G8/U9
duplex-CΨG
mod_NMR
mod
ref
Roll [°]
Base-pair Step
U1/C2
C2/A3
A3/C4
C4/Ψ5
Ψ5/G6
G6/A7
A7/G8
G8/U9
duplex-AΨU
mod
ref
Roll [°]
Base-pair Step
U1/C2
C2/A3
A3/A4
A4/Ψ5
Ψ5/U6
U6/A7
A7/G8
G8/U9
duplex-UΨA
mod
ref
Roll [°]
Base-pair Step
U1/C2
C2/A3
A3/U4
U4/Ψ5
Ψ5/A6
A6/A7
A7/G8
G8/U9

(C) Inclination (η)

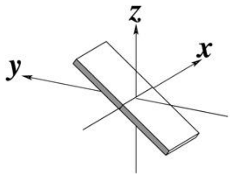

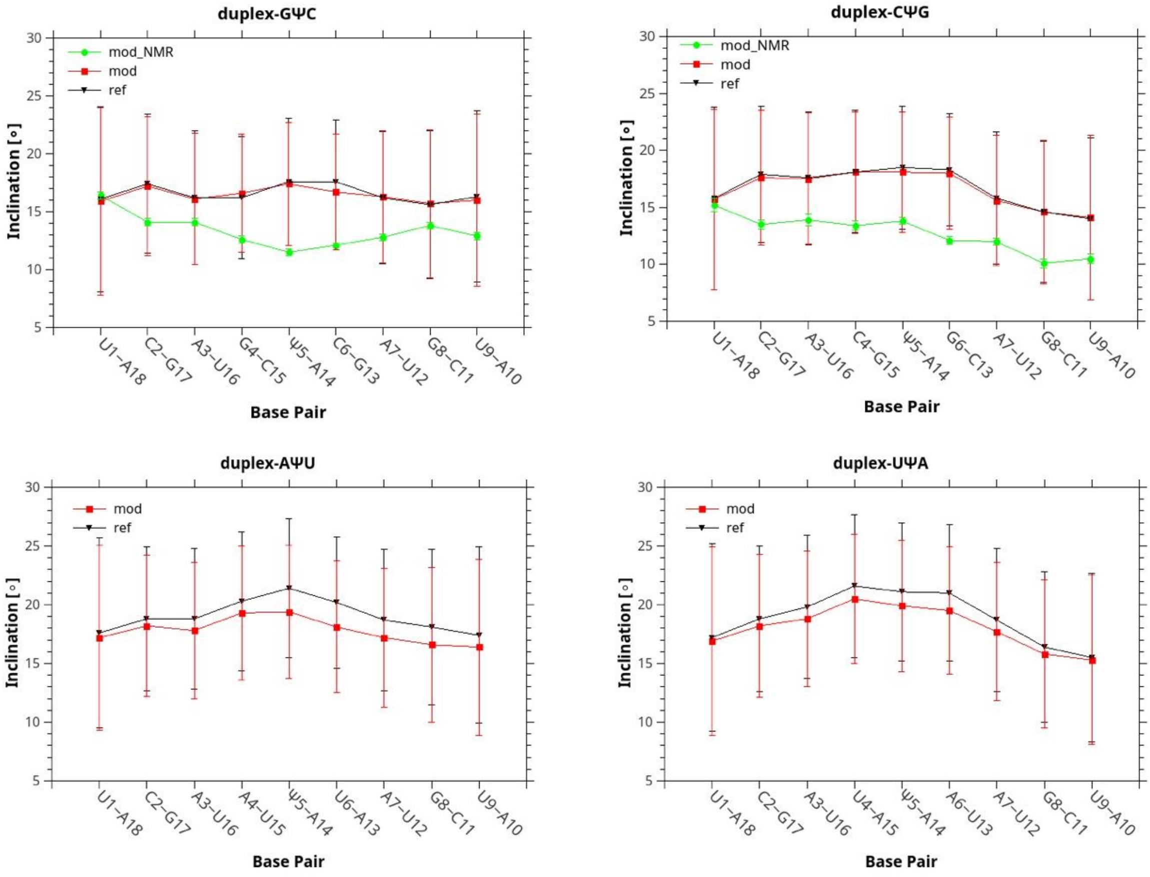

Supplementary Figure S6. Intra-strand P-P distances (Å) from 5' to 3' direction; green – Ψ-modified NMR; red – Ψ-modified from MD simulation; black – reference, unmodified from MD simulation. Vertical lines represent standard deviations.

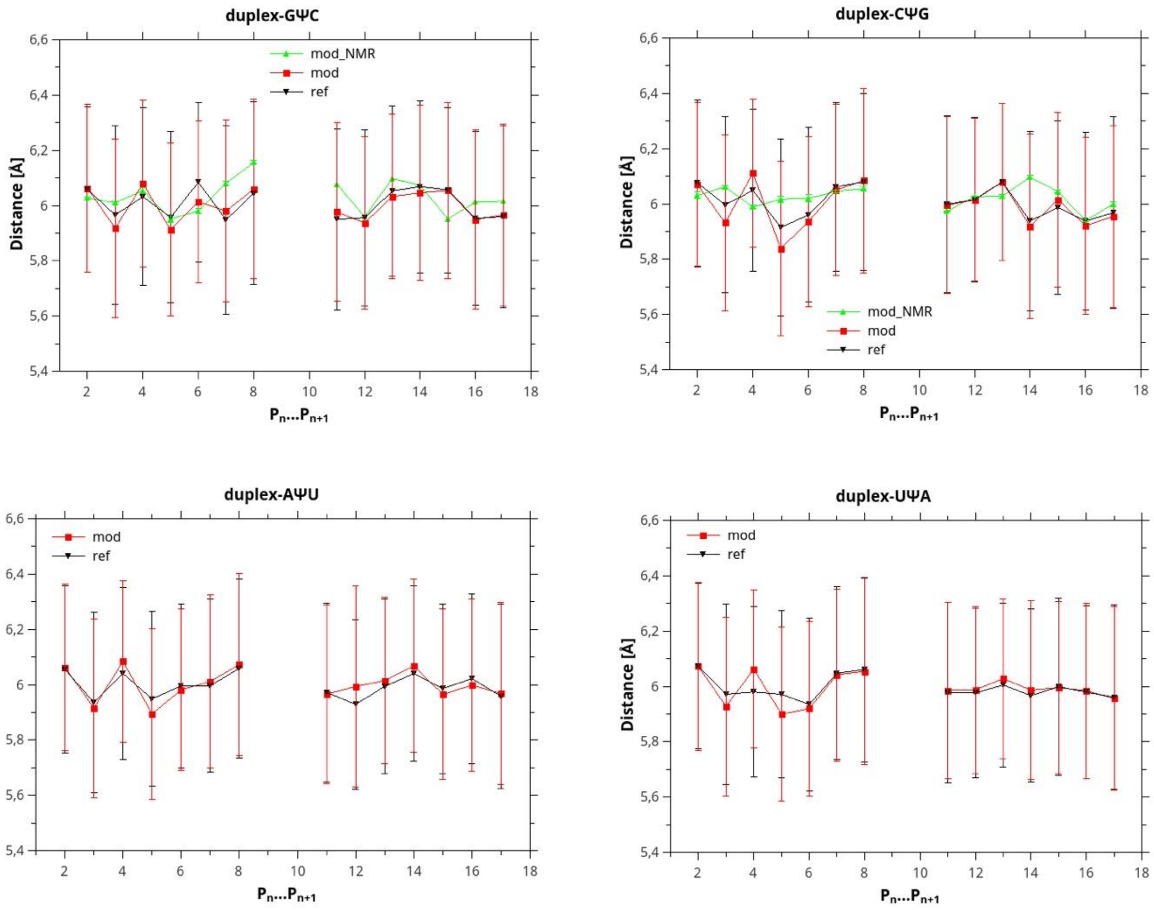

Supplementary Figure S7. Inter-strand C1'-C1' distances (Å); green – Ψ-modified NMR; red – Ψ-modified from MD simulation; black – reference, unmodified from MD simulation. Vertical lines represent standard deviations.

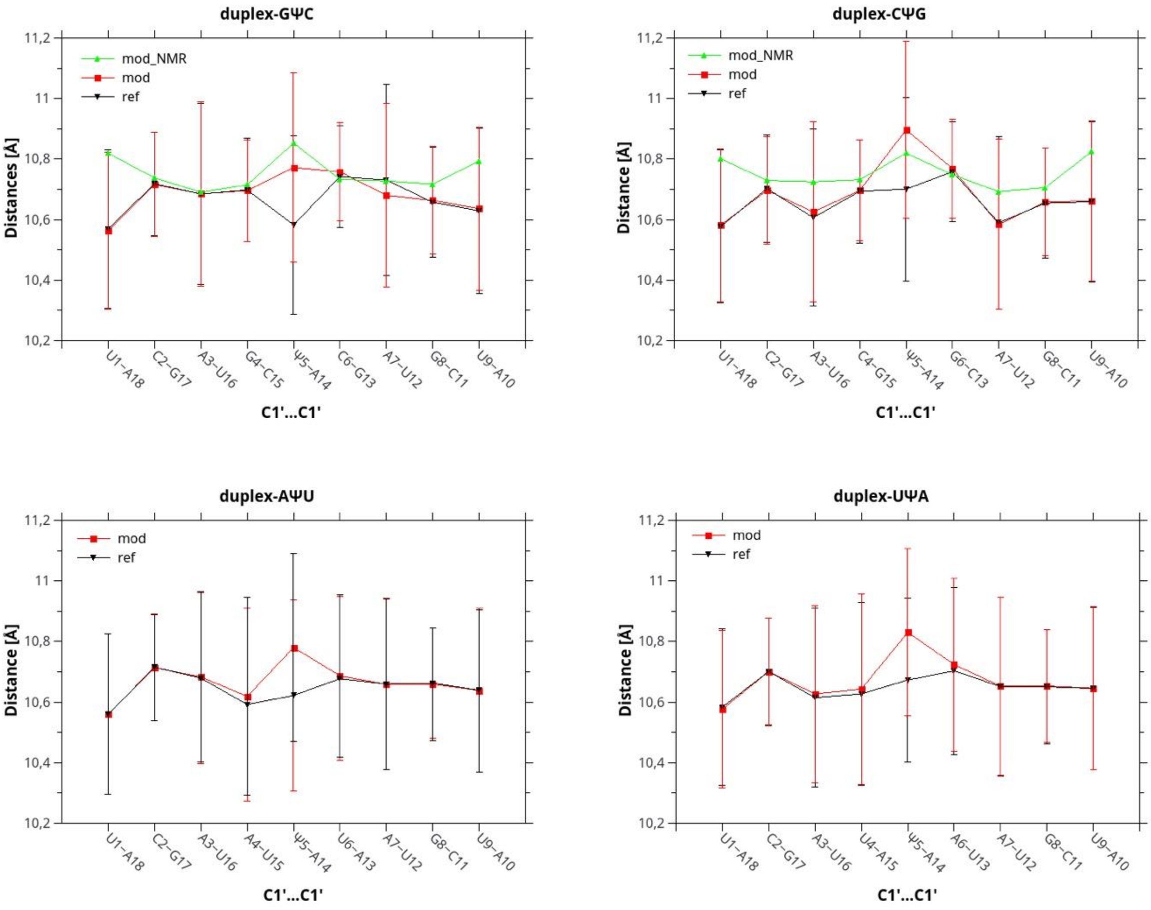

Supplementary Figure S8. Glycosidic torsion angles (χ) for Ψ5 and U5 in the simulated duplexes; red – Ψ-modified duplexes; black – reference duplexes, unmodified.

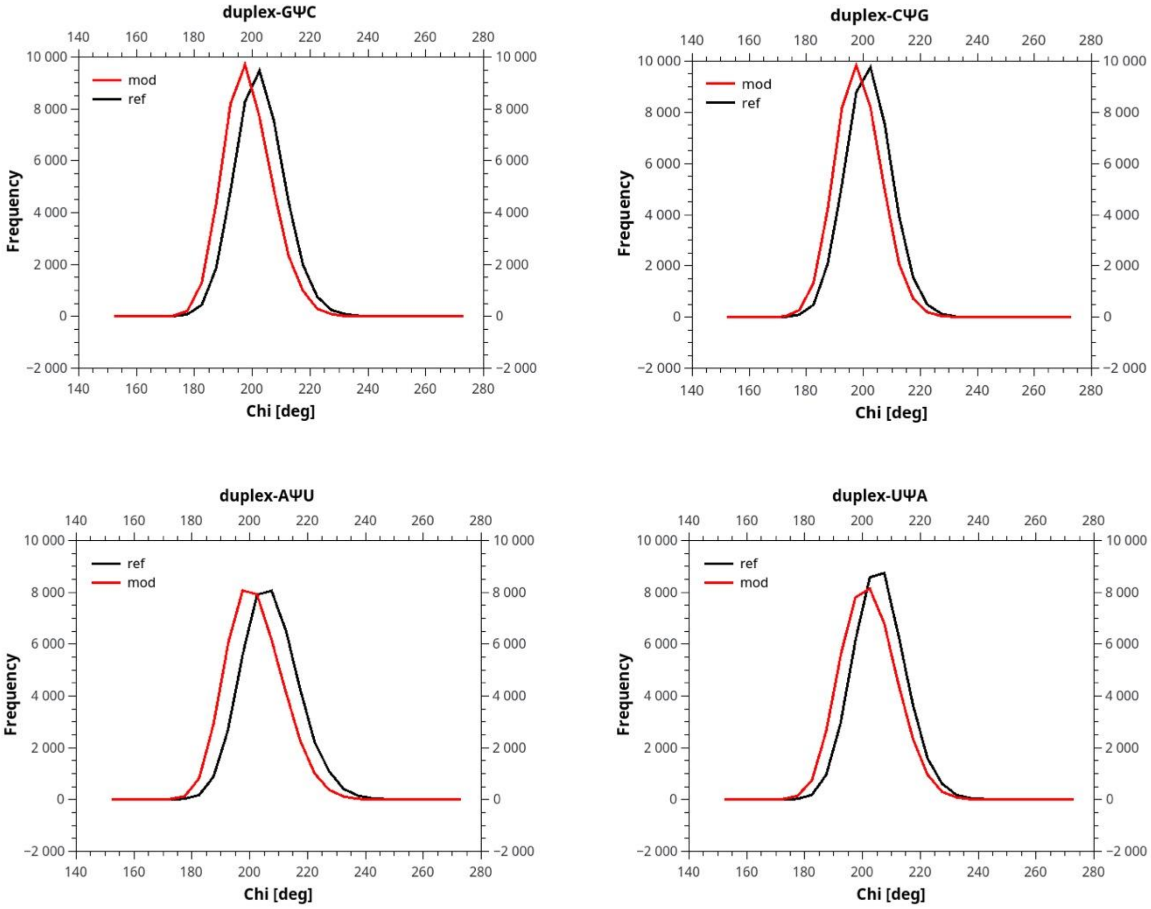

Supplementary Figure S9. View of the stacked Ψ-A base pair in 8 unique base pair steps in the studied duplexes. In each window, in the strand on the left side the 5′ base is on the top, on the left column the Ψ-A bp is in the bottom, on the right column it is in the top. The geometries are for the average structures of the most populated cluster over the MD trajectories.

Duplex-GΨC

5′GΨ/3′CA bp step	5′ΨC/3′AG bp step
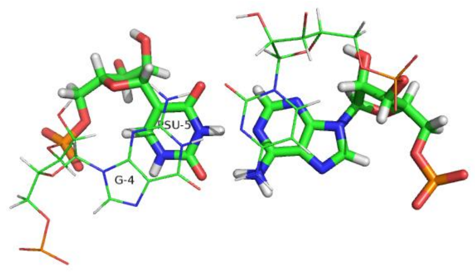	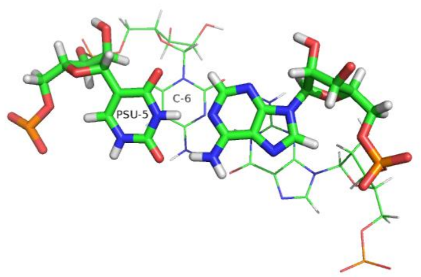

Duplex-CΨG

5′CΨ/3′GA bp step	5′ΨG/3′AC bp step
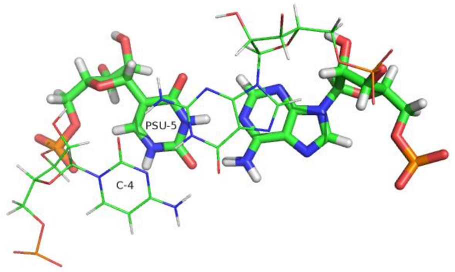	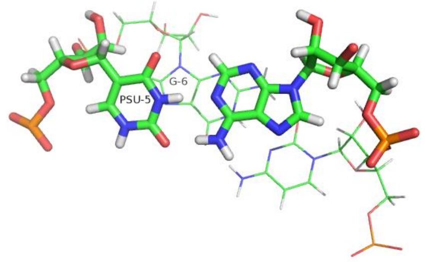

Duplex-AΨU

5′AΨ/3′UA bp step	5′ΨU/3′AA bp step
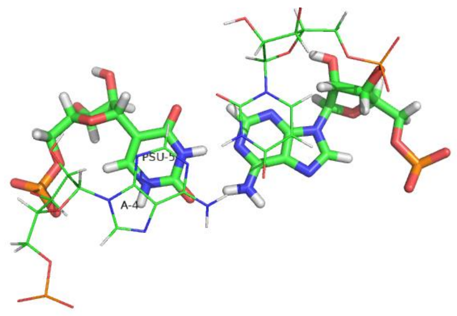	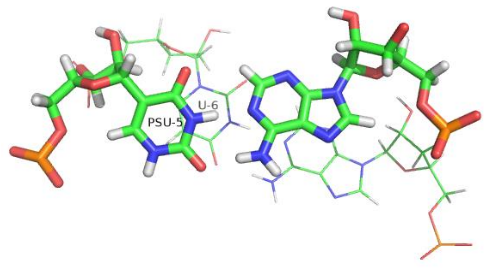

Duplex-UΨA

5′UΨ/3′AA bp step	5′ΨA/3′AU bp step
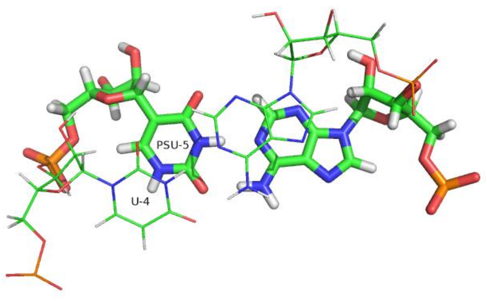	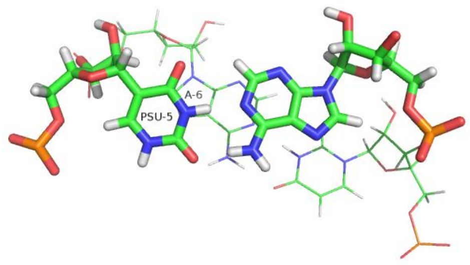

Supplementary Figure S10. QM-based stacking energies for 8 unique base pairs steps containing the Ψ-A base pair (red) and their unmodified counterparts (blue). The base pair steps are ordered according to the decreasing stability from the left to the right.

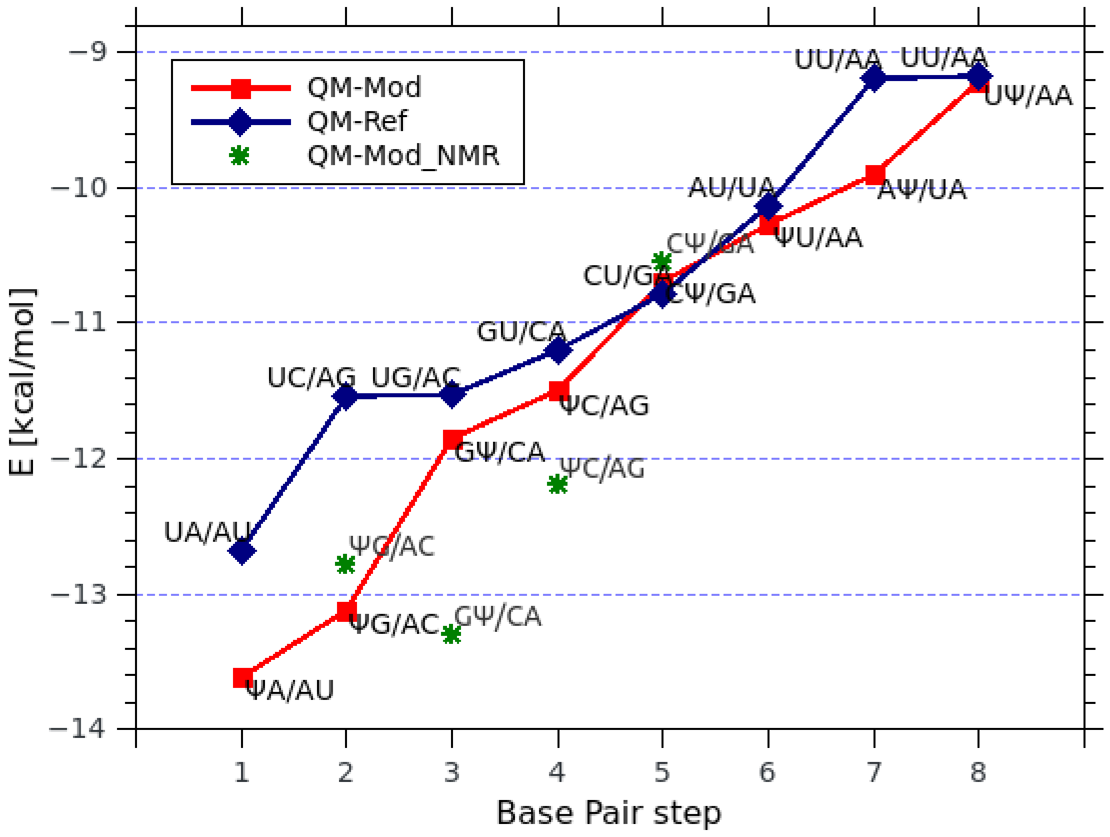

Stacking energies for the base pair steps containing the Ψ-A base pair fall within the range -13.62 kcal/mol (5′ΨA/3′AU base pair step) to -9.21 kcal/mol (5′UΨ/3′AA base pair step) whereas for the unmodified counterparts the range falls between -12.68 kcal/mol to -9.17 kcal/mol. The most stable step involving the Ψ-A base pair is the 5′ΨA/3′AU base pair step. The second most stable step is 5′ΨG/3′AC base pair step. These steps are more stable than any base pair steps with U-A base pair.

For comparison stacking energies were calculated for the geometries derived from NMR models for duplex-GΨC and duplex-CΨG. Stacking energies for the base pair steps calculated for the geometries within the same duplex follow the same trend as the energies calculated for MD-derived geometries. Stacking energies calculated for the geometries derived from NMR models of distinct duplexes shows discrepancies in the energy trend obtained for MD-derived geometries. The observed differences in base pair step stacking energies between MD and NMR geometries are quite expected and result from the fact that the NMR structures were energy minimized whereas representative geometries derived from the MD trajectories experiencing thermal fluctuations.

Supplementary Figure S11. Impact of Ψ to the QM stacking interaction energies of the base pair steps: intra-strand (blue) and inter-strand (green) contributions. ΔE = E_{modif}-$E_{unmodif}$.

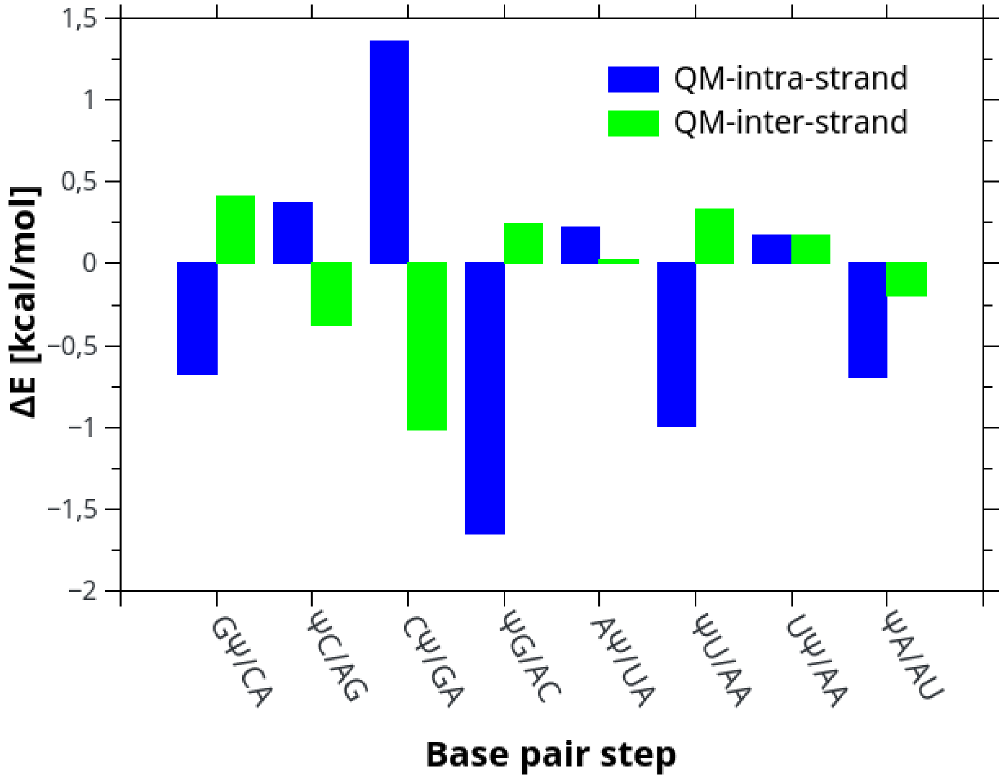

The stabilization effects of Ψ at the 5′ΨG/3′AC, 5′ΨU/3′AA and 5′GΨ/3′CA base pair steps are dominated by the more favorable intrastrand ΨG, ΨU and GΨ stacking interactions compared with those of UG, UU and GU, respectively. At these base pair steps, the Ψ slightly destabilized the interstrand interactions. At the 5′ΨA/3′AU base pair step, both the intrastrand ΨA and interstrand Ψ/U stacking interactions were found to be more favorable than those at the 5′UA/3′AU base pair step. At the 5′CΨ/3′GA base pair step, the Ψ destabilized the intrastrand stacking interactions by 1.35 kcal/mol while stabilizing the interstrand stacking by 1.0 kcal/mol. At the 5′ΨC/3′GA base pair step, the slight destabilization of 0.37 kcal/mol from the intrastrand interactions was compensated by the stabilizing interstrand interactions.

Supplementary Figure S12. Interaction energies calculated for the base pair steps in this work, based on the average geometries of the most populated clusters derived from MD simulations of unmodified duplexes in this paper (B97D/Def2TZVPP) and by Svozil et al., [6] based on the average-energy geometries (RI-DFT-D/TPSS/LP).

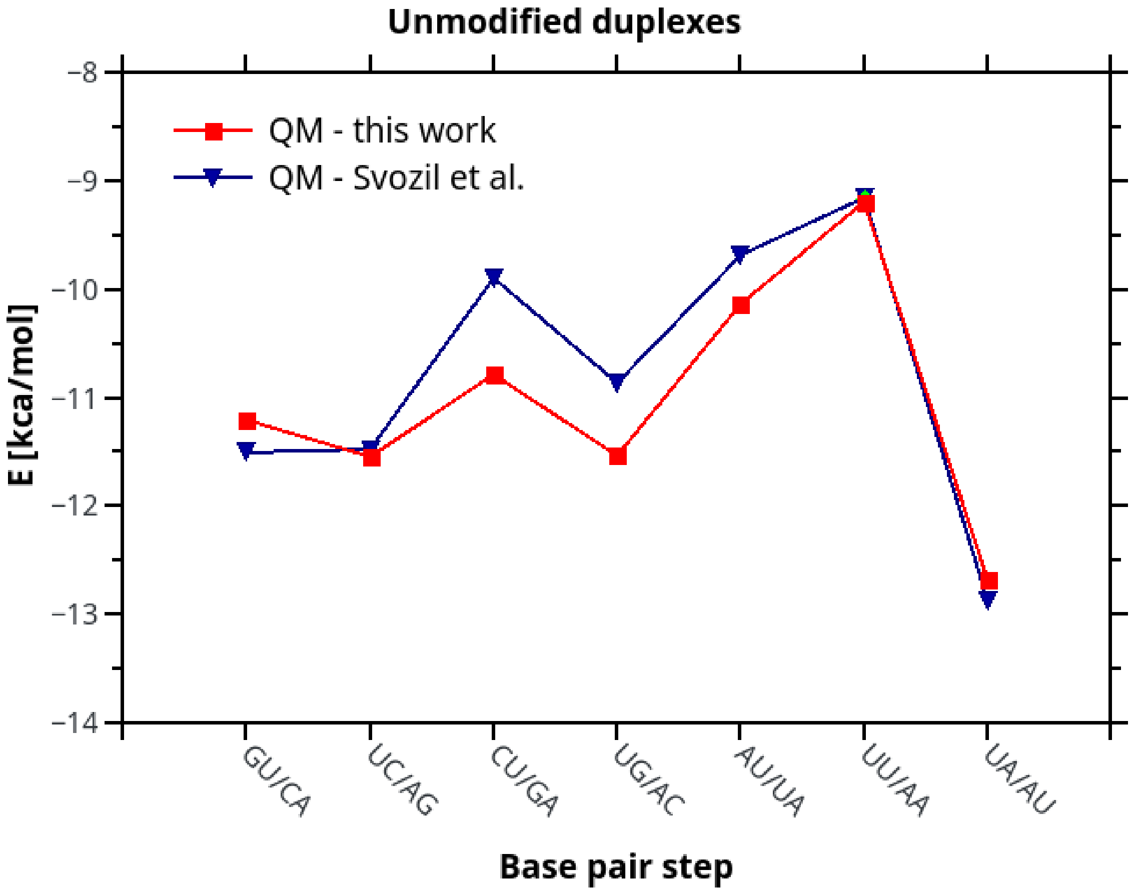

Supplementary Figure S13. Change in the stacking energy between base pairs at given base-pair step upon Ψ modification (red – QM, grey – MM). The data are ordered in decreasing stability (as calculated with QM) of 8 unique dinucleotide steps containing Ψ-A base pair in RNA duplexes. $\Delta E = E_{modif} - E_{unmodif}$.

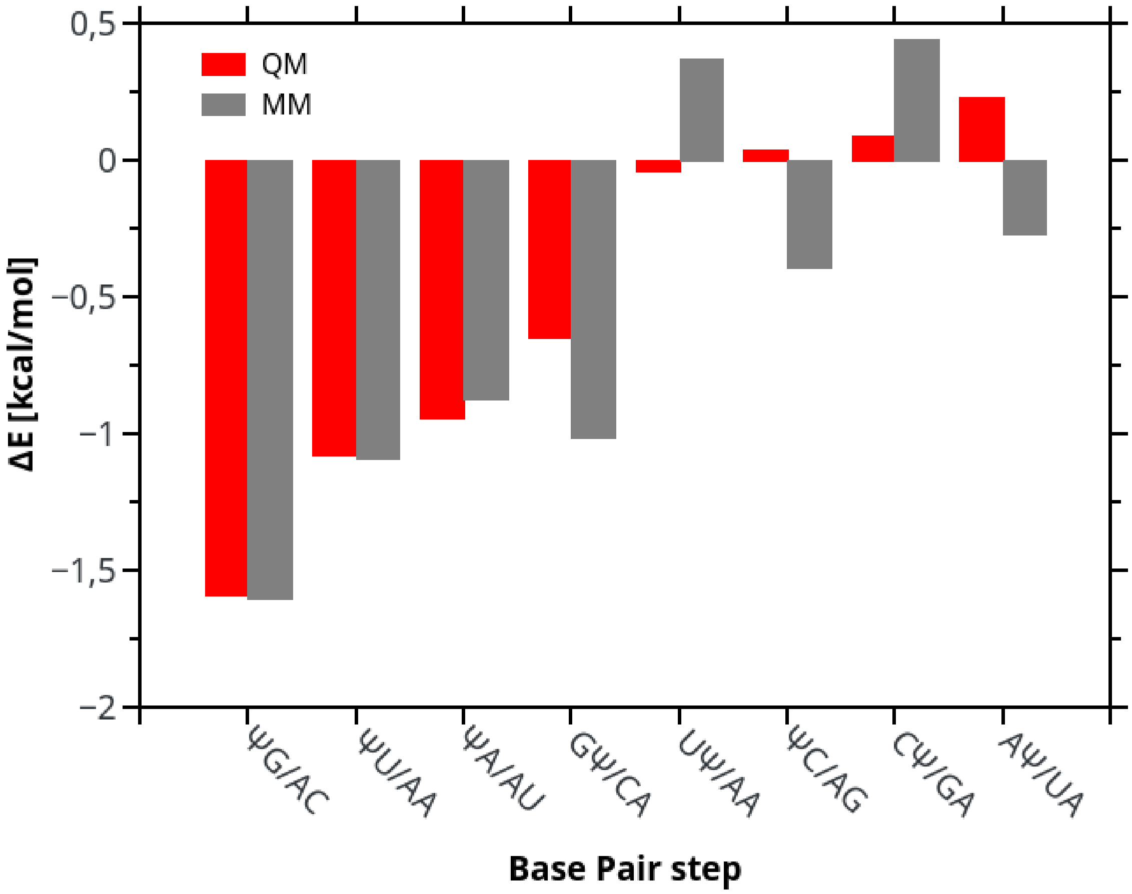

Supplementary Figure S14. Change in the stacking energy between bases in a single strand of RNA duplex at given base step (ΔE intra-strand) upon Ψ modification (red – QM, black – MM with the bars showing standard deviation). $\Delta E = E_{modif} - E_{unmodif}$.

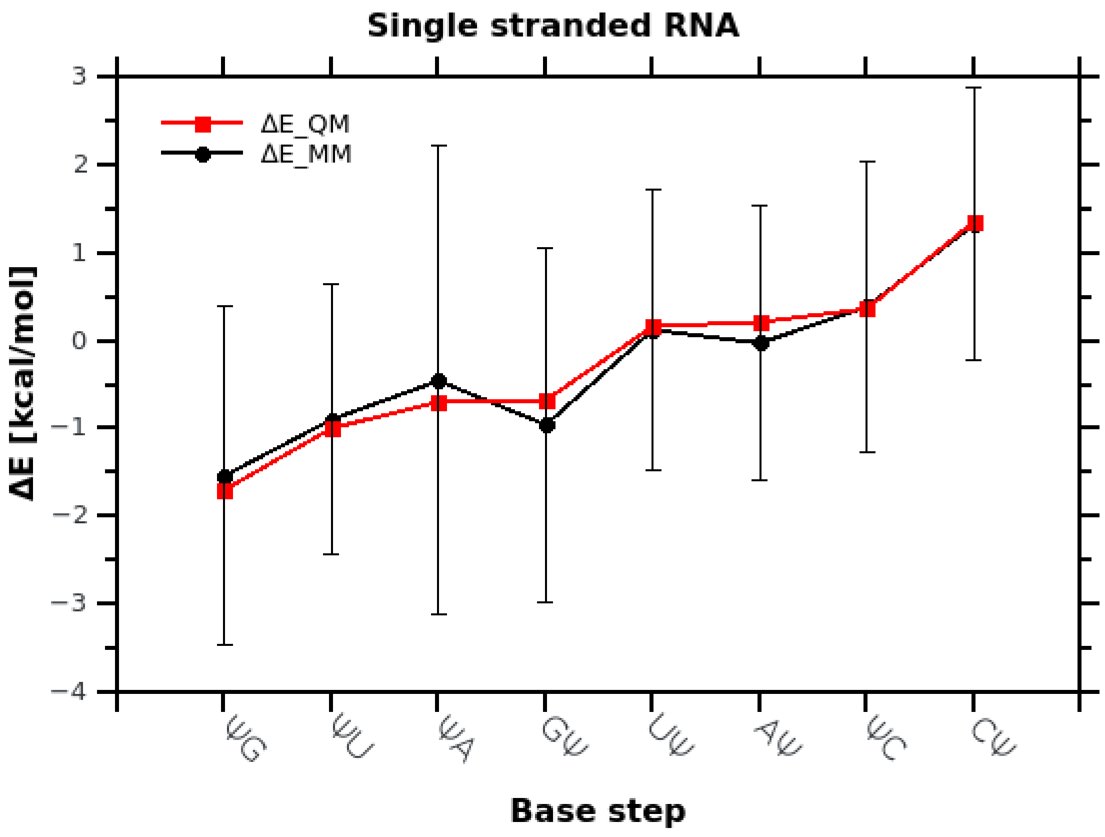

Supplementary Figure S15. Prediction of the impact of Ψ to the staking energies at trinucleotide steps; orange – ΔE_{QM}; grey – ΔE_{MM}. $\Delta E = E_{modif}-E_{unmodif}$.

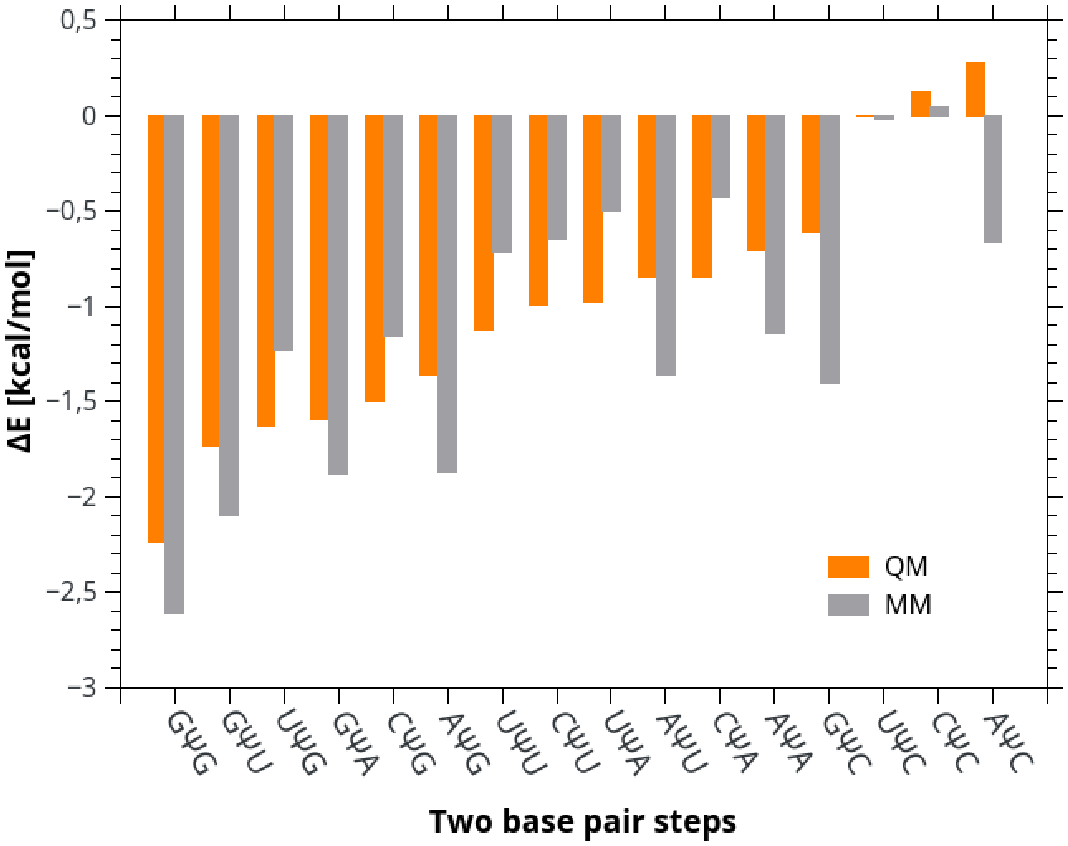

Supplementary Figure S16. Change in the QM stacking energy at two base pair steps upon Ψ modification in comparison with the experimental change in thermal stability of the duplexes (red – QM, green – experiment [5]). ΔE = E_{modif}-$E_{unmodif}$.

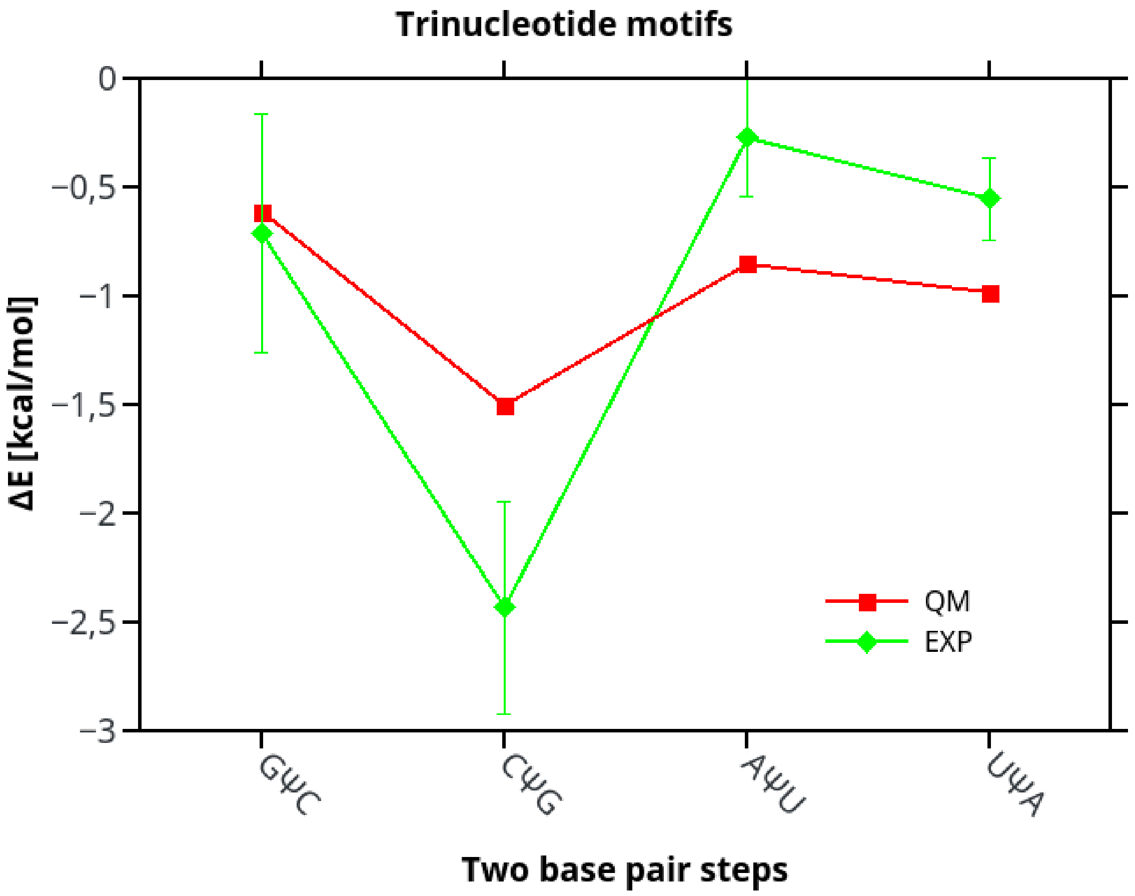

The change in the stacking energies upon replacing U with Ψ to obtain an internal Ψ-A base pair at the GΨC and CΨG steps correlates with the experimental result [5] showing a more favorable thermodynamic effect for Ψ modification of duplex-CΨG than that of duplex-GΨC (Supplementary Fig. S16). Moreover, the changes in the stacking energies upon the Ψ modifications at the AΨU and UΨA steps agree with the less favorable thermodynamic effects of Ψ modifications for duplex-AΨU and duplex-UΨA.

In general, there is no quantitative correlation between the QM gas phase stacking data and the nucleic acid stability [7,8]. The QM calculations provide intrinsic stacking energies that correspond to the gas phase environment [8,9] . The free energy effect that is associated with the base sequence includes not only intrinsic base stacking but also entropy effects and hydration. Moreover, stacking calculations are performed with respect to the no-stacking reference state, whereas thermodynamic measurements of duplex formation are performed with respect to a single strand, where some portion of stacking can be expected. Nevertheless, Johnson and coworkers [10], using nucleobase geometries from average fiber diffraction data and QM energy calculations, demonstrated a correlation between the predicted and experimental nearest-neighbor binding energies.

Supplementary Figure S17. Isodensity surface color-coded by electrostatic potential (ESP). U-A and Ψ-A base pairs (top row), internal triple base pairs in the Ψ-modified sequences (right side) and reference sequences (left side), view from the major groove side. The negative ESP regions are indicated in red, and the positive regions in blue.

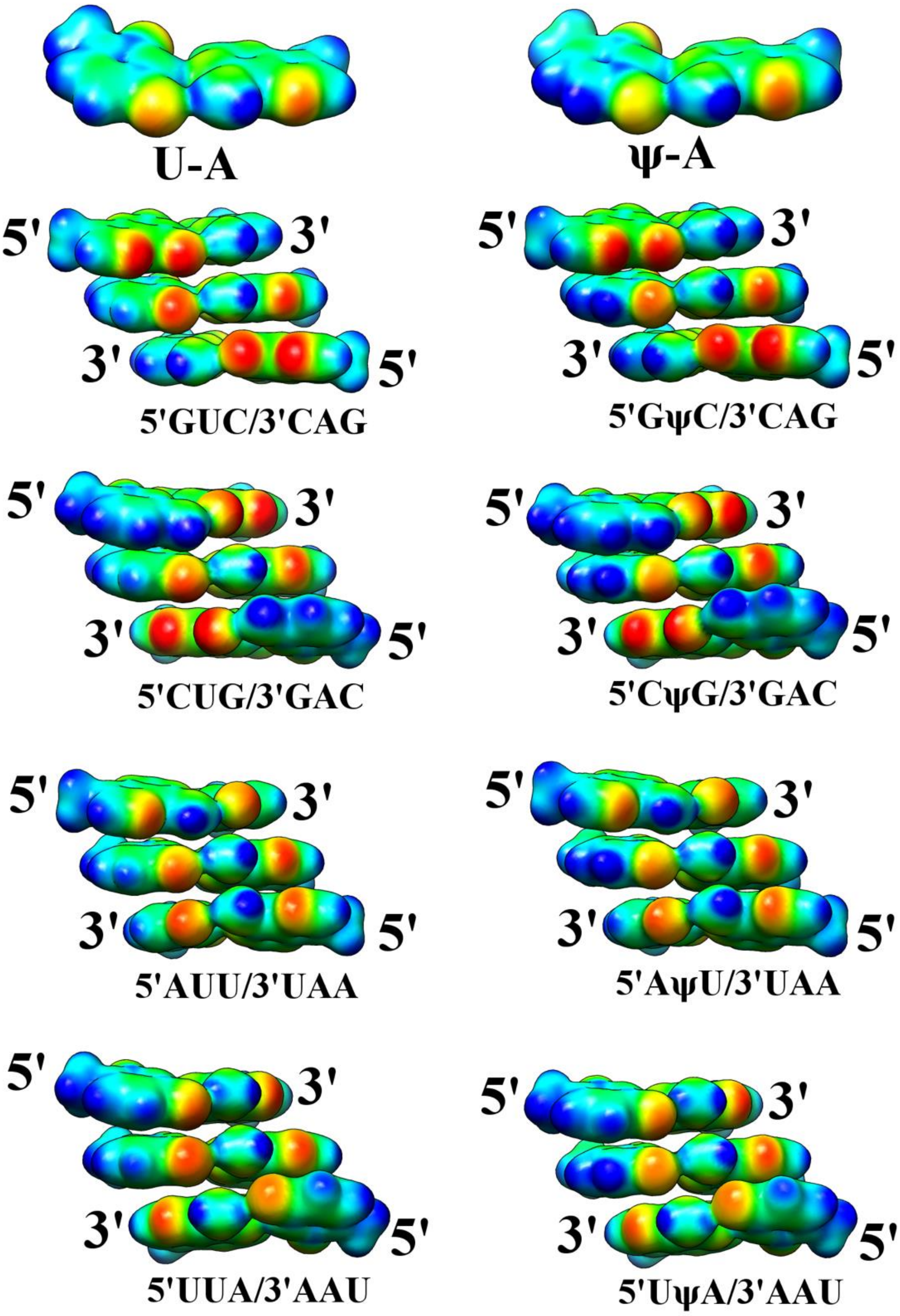

The isodensity surfaces for the U-A and Ψ-A base pairs indicated rearrangement of the charge localization around the major groove due to the isomerization of uridine to Ψ, but the isodensity surface shapes were very similar. The Hoogsteen edge of Ψ is more positive than that of U, with positive charge located on the hydrogen attached to the N1 atom. The stabilization of the stacking interactions at the 5′-GΨ/3′-CA base pair step can be rationalized by the attractive interactions due to the proximity of the electropositive region located at

HN1-Ψ and the electronegative region at N7 of G (distance 3.5 Å). The increased electropositivity of HN1-Ψ compared with that of the corresponding H5 in U also contributes to the stabilization at the 5′ΨG/3′AC and 5′ΨA/3′AU base pair steps where the HN1-Ψ…N7-A/N7-G distance is 4.1 Å and at the 5′ΨU/3′AA base pair step where the HN1-Ψ…O4-U distance is 4.4 Å. On the other hand, the electropositive regions at the H6 of cytidine and HN1-Ψ contribute to the destabilization of the interaction energy at the 5′CΨ/3′GA base pair step.

Supplementary Figure S18. Distribution of the distance between HN1-Ψ5 atom and OP2 atom of the preceding residue in the simulated Ψ-modified duplexes.

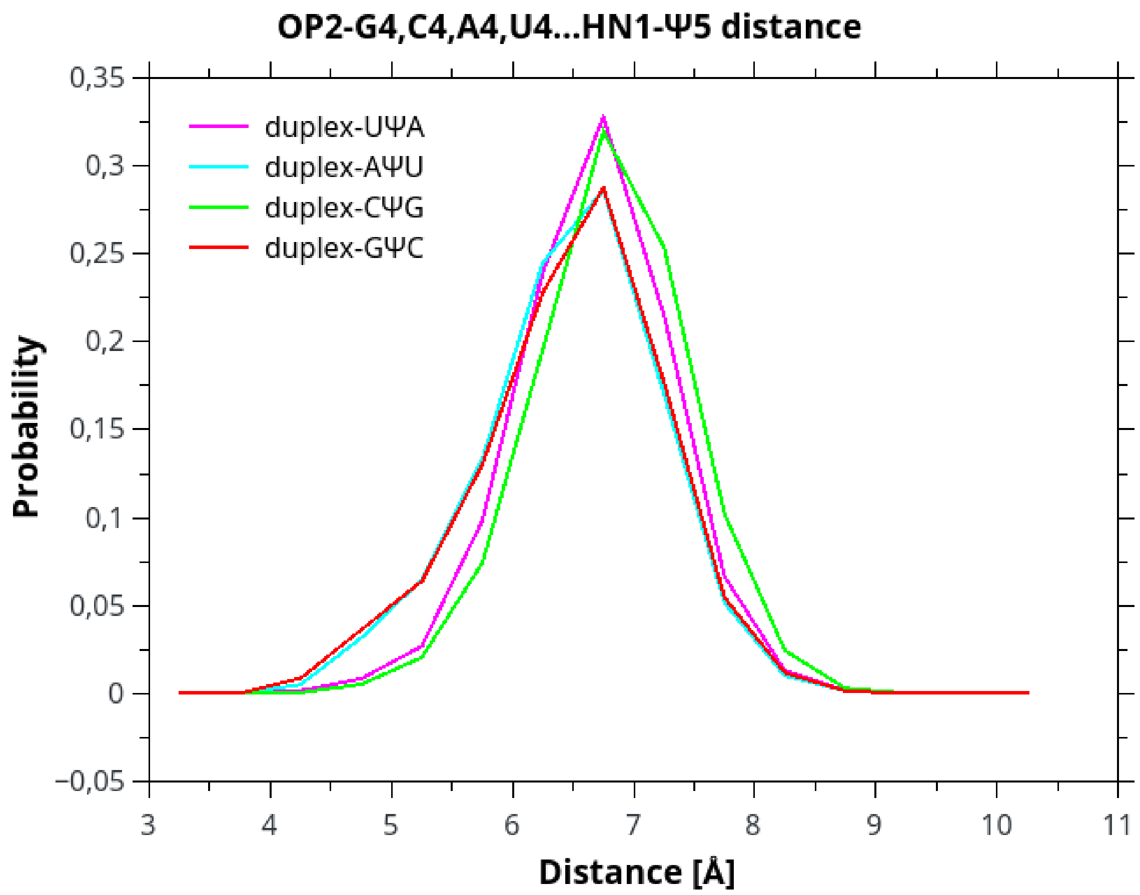

Supplementary Figure S19. Schematic representation of base pair interactions in one base pair step. Here, A, B, C and D represent the bases. The solid lines represent intra- and interstrand interaction energies, while the dotted lines represent hydrogen bonding interaction energies between the base pairs.

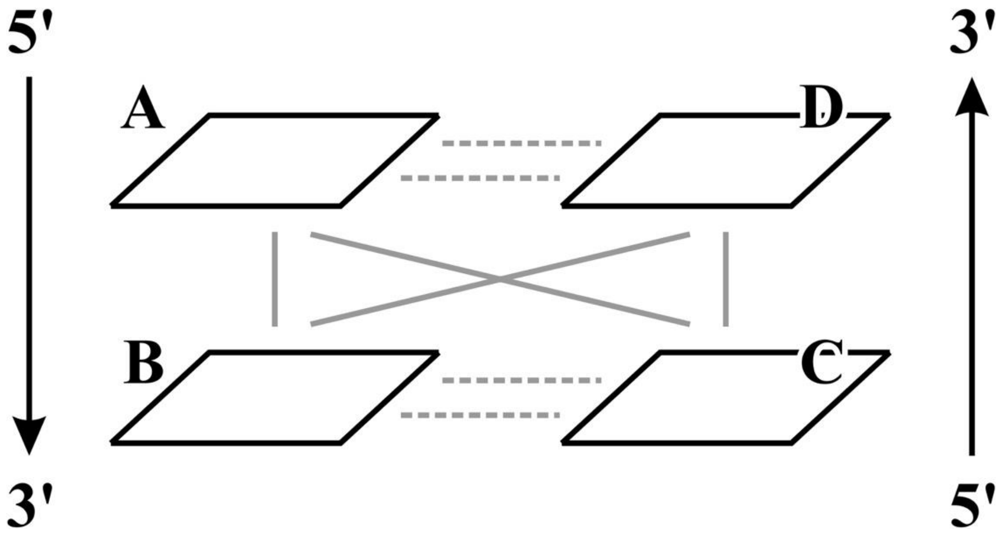

SUPPLEMENTARY METHODS

1. *RNA Synthesis*

For NMR study, each of the obtained duplexes was dissolved in a volume of 200 µl of 90% H_2O and 10% D_2O solution and placed into 3 mm NMR sample tubes. Buffer conditions were 10 mM sodium phosphate, 150 mM NaCl, 0.1 mM EDTA at pH 6.8. For experiments performed in D_2O, the samples were evaporated to dryness from D_2O three times and finally redissolved in 99.996% D_2O. To allow a hybridization of the strands to yield the duplex before NMR measurements or after water exchange each sample was heated for 3-5 min at 90 °C and then slowly cooled to room temperature.

2. *Structural Restraints*

Watson-Crick base pairs and hydrogen bonding pattern between Ψ and A bases were identified according to [5] based on observation of downfield shifted imino and amino proton resonances and characteristic NOE correlations. Thus, restraints for hydrogen bonds taken from the standard base-pair geometries of nucleic acids as suitable distances with a tolerance of ±0.2 Å were also imposed. The constraints for endocyclic and backbone torsion angles were obtained on the basis of the estimates of $^3J_{H\text{-}H}$ couplings as described previously [11,12] . The sugar pucker conformation was constrained to 3′-*endo* for most of residues due to lack or weak visible H1′ to H2′ cross-peaks in the ^{1}H-^{1}H COSY spectra. Thus ranges for all endocyclic ν_0-ν_4 torsion angles were set as follows $\nu_0 = 3 \pm 15°$, $\nu_1 = -25 \pm 15°$, $\nu_2 = 37 \pm 15°$, $\nu_3 = -36 \pm 15°$, $\nu_4 = 21 \pm 15°$ except torsions of the U9 and A18 residues at the 3′-ends of the polynucleotide chains. In case of the latter ones, intermediate $^3J_{H1'\text{-}H2'}$ couplings were observed and to reflect the possibility of conformational averaging sugar puckers were left unconstrained. The orientations of the nucleobases with respect to the sugar rings characterized by the glycosidic torsion angles (χ) were derived by the intensity of the intranucleotide H1′-H6/H8 cross-peaks in 2D NOESY spectra and accordingly restrained to allow *anti* range (-158 ± 30°). The backbone α and ζ were set to 0 ± 120° to exclude the *trans* conformation upon the ^{31}P chemical shifts analysis (Varani et al. 1996). An estimation of $^3J_{P\text{-}H3'}$, $^3J_{P\text{-}H5'}$, $^3J_{P\text{-}H5''}$ in ^{1}H-^{13}P COSY spectra provided information on backbone β and ε angles. According to the observations torsion angles β were restrained to the *trans* conformation (178 ± 30°) for both duplexes. Similarly the ε angles were restricted to the typical for A-form helices *trans* range of -153 ± 30°. Taking into regard H4′-H5′/H5″ couplings observed in the ^{1}H-^{1}H COSY spectra the γ torsion angles were restricted to a *gauche*$^+$ range (54 ± 30°).

3. *Structure calculations by simulated annealing method*

The starting structures were energy minimized with 50 steps of steepest descent followed by 150 steps of conjugate gradient algorithms in an implicit solvent environment using the default radii (igb = 1) and the *sander* module. A cutoff of 12 Å was chosen to include the nonbonded interactions during energy minimization. Different initial velocities were assigned

for the 100 independent restrained MD (rMD) simulations during the first cycle. The trajectories were propagated using Langevin dynamics with an absolute value of 20.0 for any component of the velocity vector, a time step of 1 fs and a collision frequency of 1 ps^{-1}, applying constraints to the bonds involving hydrogen atoms by the SHAKE algorithm. A maximum distance cutoff of 12 Å between the atom pairs was used for the pairwise summation involved in calculating the effective Born radii, while to include the nonbonded interactions, a long-range cutoff of 20 Å was considered during the restrained MD simulations. The temperature of the system was raised from 0 K to 3000 K within the first 5 ps and was held constant at 3000 K for the next 3 ps. Then, the system was cooled to 100 K within the next 82 ps, followed by further cooling to 0 K within the next 10 ps. All the NOE distances and dihedral angles around the backbone that were associated with sugar puckering were restrained by force constants of 12 kcal/mol/$Å^2$ and 12 kcal/mol/rad^2, respectively, using a potential well with a flat bottom with parabolic sides to make sure that each violation was active. Chirality restraints were also applied by a force constant of 12 kcal/mol/rad^2 to restrain the orientations of the chiral atoms by restricting the flipping of covalent bonds at high temperature. We followed a similar simulated annealing protocol for the second cycle with the exceptions that the temperature was raised to 600 K, and the force constants applied for the NOE distance restraints and dihedral and chirality restraints were 30 kcal/mol/$Å^2$ and 30 kcal/mol/rad^2, respectively. In both of the simulated annealing cycles, the weight of the restraints varied from 0.1 to 1.0 within the first 8 ps of heating and equilibration to allow large violations and thereafter was held at 1.0 for the rest of the 92 ps of simulation time.

4. *Molecular Dynamics (MD) Simulations*

The initial model build structures was solvated with TIP3P [13] water molecules in truncated octahedral boxes in a manner so that the closest distance between any atom of the solute molecule and the edge of the periodic box is 10 Å. All the systems were neutralized with Na^+ ions and additional Na^+ and Cl^- ions, using Joung and Cheatham ion parameters [14] , were added to the neutralized and solvated systems to set the salt concentration at 1.0 M. This salt concentration intended to replicate the environment of UV melting experiments [5].The influence of ion concentration in the MD simulations on RNA structure and dynamics has been discussed elsewhere [15,16]. The Na^+ and Cl^- ions were swapped with random water molecules that were not closer than 6.0 Å from any residue of the 9-bp duplexes and not closer than 4.0 Å from any other Na^+/Cl^- ions. The solvated systems of both the unmodified and Ψ-modified RNA duplexes were subjected to energy minimization by 200 steps of steepest decent followed by 300 steps conjugate gradient with a weak RMS force convergence criteria of 0.1 kcal/mol for the energy gradient holding the duplexes by a positional restraint force of 25.0 kcal/mol/$Å^2$. In the next step, constant pressure Langevin dynamics using a collision frequency of 1 ps^{-1} was performed for 50 ps at 300 K to equilibrate the solvent molecules holding the duplexes by a positional restraint force of 25.0 kcal/mol/$Å^2$. Then the systems were further subjected to two stages of energy minimizations by 500 steps of steepest decent followed by 2000 steps conjugate gradient in each stage and with the default RMS force convergence criteria of 0.00010 kcal/mol with the exception in the first stage that a positional restraint force of 25.0 kcal/mol/$Å^2$ was applied to the duplexes. These two stages of

energy minimizations were followed by three stages of equilibration before production run. In stage 1, the systems were slowly heated from 0 K to 300 K in 80 ps using constant volume Langevin dynamics with a collision frequency of 5 ps^{-1} holding the duplexes with a restraint force of 15 kcal/mol/$Å^2$. The temperature was held constant at 300 K for the next 20 ps. In the second stage of 100 ps equilibration run at 300 K, constant pressure Langevin dynamics with a collision frequency of 1 ps^{-1} was used holding the duplexes with a lower restraint force of 10 kcal/mol/$Å^2$. In the final stage of equilibration run for 1 ns at 300 K, the conditions of previous stage were followed except the positional restraint force on the duplex molecule was removed and to avoid fraying ends the inter-strand Watson-Crick hydrogen bonding distance restraints were applied to the terminal base pairs following Saenger [17] which allowed 0.1 Å movement from the equilibrium bond distance. The conditions for the production run were similar to the final stage of the three stage equilibration. Trajectory files were written at each 10 ps time step during a total 500 ns of production run. Constant pressure simulations were carried out using Berendsen barostat [18] by turning on isotropic position scaling with a reference pressure of 1 atm and a pressure relaxation time of 2 ps. The nonbonded interactions within 10.0 Å long-range cutoff were taken into account during minimizations, equilibrations and production run. All the equilibration and production MD simulations were run with a 2 fs time step. SHAKE [19] was turned on for bonds involving hydrogen atoms. The particle mesh Ewald (PME) method [20] with a direct space cutoff of 10 Å was used for all the simulations.

5. *Analysis of MD trajectories*

The last 400 ns of trajectories from the total 500 ns of production runs were analyzed. During *lie* analysis, we considered a cutoff of 999.0 Å for both the electrostatic and van der Waals interactions. Cluster analysis was carried out with the initial clustering for every 10 frames only, the number of clusters was set to 5 and the minimum distance between clusters greater than 2.0 Å. For cluster analysis, the distance metric used was the coordinate RMSD of the heavy atoms of residues 3-7, 12-16. The most populated clusters contained of 60-90% frames. The MM-PBSA python script included in AMBER was used for the calculations of free energy (ΔG) of duplex formation. For MM-PBSA calculations, we used single trajectory approach and a total of 8000 frames from the last 400 ns trajectories and an ionic strength of 150 mM were considered. For S^2 order parameter calculation, the C1'-H1' bond [21] was chosen as *ired* vector to define and diagonalize *ired* matrix. Second order Legendre polynomials was used to determine spherical harmonics and 10 ps timestep was chosen with a maximum time 10 ns to calculate the correlation function. The correlation functions were calculated using direct method and were normalized.

6. *Ab Initio Base Pair Step Stacking Energies*

The sugar-phosphate backbone was removed from the selected geometries, and the bases were replaced by the monomers of the bases optimized individually in the gas phase at the B97D/Def2TZVPP level of theory. For each monomer of the base, the N1/N9-C1'/C5-C1' bond was replaced by the N1/N9-CH3/C5-CH3 bond. For the geometry optimization

calculations of monomers of the bases, the structures were prepared using the molecular structure editor MOLDEN [22]. The planarity of the exocyclic -NH_2 groups was maintained by freezing the dihedral angle associated with the groups during the geometry optimization calculations.

Choice of proper geometries for stacking calculations

The QM-based stacking energies have previously been shown to be sensitive to the geometry [6]. Such geometry, in principle, can be derived from several sources such as fiber diffraction data, high resolution X-ray structures and molecular dynamics (MD) simulations. Svozil and coworkers proposed a protocol for the selection of geometries based on MD simulations that are the average-energy geometries [6]. In our calculations, we used average geometries derived from the cluster analysis of the trajectories [23]. To ascertain that our base pair step geometries represent the systems sufficiently well, we compared the stacking energies of eight unique base pair steps in the unmodified duplexes calculated for our geometries with those evaluated by Svozil et al. at the average-energy geometries (Supplementary Fig. S12). The plot shows the same trends for the sequence dependences of the stacking energies calculated with both methods.

Additionally, we have calculated the base pair step stacking energies of NMR-derived geometries (model 1 of ten lowest-energy solution NMR ensemble) for both the duplex-GΨC and duplex-CΨG, and compared with the base pair step stacking energies of the representative geometries derived from MD trajectories (Supplementary Figure S10).

SUPPLEMENTARY REFERENCES

1. Arnott, S., Hukins, D. W. L., Dover, S. D., Fuller, W. & Hodgson, A. R. Structures of synthetic polynucleotides in the A-RNA and A′-RNA conformations: X-ray diffraction analyses of the molecular conformations of polyadenylic acid polyuridylic acid and polyinosinic acid · polycytidylic acid. *J. Mol. Biol.* **81,** (1973).
2. Faustino, I., Pérez, A. & Orozco, M. Toward a consensus view of duplex RNA flexibility. *Biophys. J.* **99,** 1876–1885 (2010).
3. Olson, W. K., Esguerra, M., Xin, Y. & Lu, X. J. New information content in RNA base pairing deduced from quantitative analysis of high-resolution structures. *Methods* **47,** 177–186 (2009).
4. Kailasam, S., Bhattacharyya, D. & Bansal, M. Sequence dependent variations in RNA duplex are related to non-canonical hydrogen bond interactions in dinucleotide steps. *BMC Res. Notes* **7,** 83 (2014).
5. Kierzek, E. *et al.* The contribution of pseudouridine to stabilities and structure of RNAs. *Nucleic Acids Res.* **42,** 3492–3501 (2014).
6. Svozil, D., Hobza, P. & Šponer, J. Comparison of intrinsic stacking energies of ten unique dinucleotide steps in A-RNA and B-DNA duplexes. Can we determine correct order of stability by quantum-chemical calculations? *J. Phys. Chem. B* **114,** 1191–1203 (2010).
7. Šponer, J., Morgado, C. A. & Svozil, D. Comment on "Computational Model for Predicting Experimental RNA and DNA Nearest-Neighbor Free Energy Rankings". *J. Phys. Chem. B* **116,** 8331–8332 (2012).
8. Šponer, J. *et al.* How to understand quantum chemical computations on DNA and RNA systems? A practical guide for non-specialists. *Methods* **64,** 3–11 (2013).
9. Šponer, J. *et al.* Nature and magnitude of aromatic base stacking in DNA and RNA: Quantum chemistry, molecular mechanics, and experiment. *Biopolymers* **99,** 978–988 (2013).
10. Johnson, C. A. *et al.* Computational Model for Predicting Experimental RNA and DNA Nearest-Neighbor Free Energy Rankings. *J. Phys. Chem. B* **115,** 9244–9251 (2011).
11. Popenda, L., Adamiak, R. W. & Gdaniec, Z. Bulged adenosine influence on the RNA duplex conformation in solution. *Biochemistry* **47,** 5059–5067 (2008).
12. Popenda, L., Bielecki, L., Gdaniec, Z. & Adamiak, R. W. Structure and dynamics of adenosine bulged RNA duplex reveals formation of the dinucleotide platform in the C:G-A triple. *Arkivoc* **2009,** 130–144 (2009).
13. Jorgensen, W. L., Chandrasekhar, J., Madura, J. D., Impey, R. W. & Klein, M. L. Comparison of simple potential functions for simulating liquid water. *J. Chem. Phys.* **79,** 926–935 (1983).
14. Joung, I. S. & Cheatham, T. E. Determination of alkali and halide monovalent ion parameters for use in explicitly solvated biomolecular simulations. *J. Phys. Chem. B* **112,** 9020–9041 (2008).
15. Fischer, N. M., Polěto, M. D., Steuer, J. & Van Der Spoel, D. Influence of Na + and Mg 2+ ions on RNA structures studied with molecular dynamics simulations. *Nucleic Acids Res.* **46,** 4872–4882 (2018).
16. Sponer, J. *et al.* RNA structural dynamics as captured by molecular simulations: A comprehensive overview. *Chemical Reviews* **118,** 4177–4338 (2018).
17. Saenger, W. *Principles of Nucleic Acid Structure*. *RNAbook* (1984). doi:10.1007/978-1-4612-5190-3
18. Berendsen, H. J. C., Postma, J. P. M., Van Gunsteren, W. F., Dinola, A. & Haak, J. R. Molecular dynamics with coupling to an external bath. *J. Chem. Phys.* **81,** 3684–3690

(1984).
19. Ryckaert, J. P., Ciccotti, G. & Berendsen, H. J. C. Numerical integration of the cartesian equations of motion of a system with constraints: molecular dynamics of n-alkanes. *J. Comput. Phys.* **23,** 327–341 (1977).
20. Darden, T., York, D. & Pedersen, L. Particle mesh Ewald: An N·log(N) method for Ewald sums in large systems. *J. Chem. Phys.* **98,** 10089–10092 (1993).
21. Juneja, A., Villa, A. & Nilsson, L. Elucidating the relation between internal motions and dihedral angles in an RNA hairpin using molecular dynamics. *J. Chem. Theory Comput.* **10,** 3532–3540 (2014).
22. Schaftenaar, G. & Noordik, J. H. Molden: A pre- and post-processing program for molecular and electronic structures. *J. Comput. Aided. Mol. Des.* **14,** 123–134 (2000).
23. Chawla, M., Autiero, I., Oliva, R. & Cavallo, L. Energetics and Dynamics of the Non-Natural Fluorescent 4AP:DAP Base Pair. *Phys. Chem. Chem. Phys.* (2018). doi:10.1039/C7CP07400J
